# RAD51 is essential for spermatogenesis and male fertility in mice

**DOI:** 10.1038/s41420-022-00921-w

**Published:** 2022-03-15

**Authors:** Junchao Qin, Tao Huang, Jing Wang, Limei Xu, Qianli Dang, Xiuhua Xu, Hongbin Liu, Zhaojian Liu, Changshun Shao, Xiyu Zhang

**Affiliations:** 1 Key Laboratory of Experimental Teratology, Ministry of Education, Department of Medical Genetics, School of Basic Medical Sciences, Cheeloo College of Medicine, Shandong University, Jinan, China; 2 Center for Reproductive Medicine, Cheeloo College of Medicine, Shandong University, Jinan, China; 3 Department of Cell Biology, School of Basic Medical Sciences, Cheeloo College of Medicine, Shandong University, Jinan, China; 4 Department of Reproductive Medicine, Second Hospital of Hebei Medical University, Shijiazhuang, China; 5 Institutes for Translational Medicine, State Key Laboratory of Radiation Medicine and Protection, Soochow University, Suzhou, China

**Keywords:** Germline development, Cell division

## Abstract

The recombinase RAD51 catalyzes the DNA strand exchange reaction in homologous recombination (HR) during both mitosis and meiosis. However, the physiological role of RAD51 during spermatogenesis remains unclear since RAD51 null mutation is embryonic lethal in mice. In this study, we generated a conditional knockout mouse model to study the role of RAD51 in spermatogenesis. Conditional disruption of RAD51 in germ cells by Vasa-Cre led to spermatogonial loss and Sertoli cell-only syndrome. Furthermore, tamoxifen-inducible RAD51 knockout by UBC-Cre^ERT2^ confirmed that RAD51 deletion led to early spermatogenic cells loss and apoptosis. Notably, inducible knockout of RAD51 in adult mice caused defects in meiosis, with accumulated meiotic double-strand breaks (DSBs), reduced numbers of pachytene spermatocytes and less crossover formation. Our study revealed an essential role for Rad51 in the maintenance of spermatogonia as well as meiotic progression in mice.

## Introduction

Spermatogenesis is a complex process involving mitotic cell division, meiosis, and the process of spermiogenesis [1]. In the mouse, the first wave of spermatogenesis begins shortly after birth when gonocytes resume mitotic division [2]. Gonocytes give rise to either undifferentiated spermatogonia or differentiating spermatogonia, which differentiate into meiotic spermatocytes [3]. Spermatocytes undergo two meiotic divisions to form haploid round spermatids, which are transformed into spermatozoa [4] Meiosis is an essential step of spermatogenesis, during prophase I of meiosis, homologous chromosomes pair, synapse, and exchange their genetic material through homologous recombination (HR) [5].

HR is essential for high-fidelity DNA repair during mitotic proliferation and meiosis [6]. Mitotic HR promotes genome stability through the precise repair of DNA double-strand breaks (DSBs) [7]. Meiotic HR is a specialized process that involves homologous chromosome pairing and strand exchange to guarantee proper chromosome segregation and genetic diversity [8]. Meiotic recombination is initiated by DSBs generated by the topoisomerase like DNA transesterase SPO11 [9]. The dynamics of γH2AX distribution is one of the major characteristics of meiosis and is commonly used as a marker for meiotic progression [10].

The repair of meiotic DSBs by HR assures the exchange of genetic material between parental haplotypes in the germline [11]. ATM kinase is needed to limit the number of meiotic DSBs and is required for complete DNA repair [12]. ATM is also essential for proper crossover formation in mouse spermatocytes [13]. After DSB formation in meiotic chromosomes, the ends surrounding the breaks are resected to create 3′ single-stranded DNA (ssDNA) overhangs [14]. The emerging ssDNA is bound and protected by heterotrimeric replication protein A (RPA) to prevent degradation and secondary structure formation [15]. Subsequently, RAD51 and DMC1 are recruited and promote the removal of RPA from the ssDNA and facilitate the invasion of 3′-extended strand into the duplex of the homologue for successful recombination and synapsis formation [16, 17].

RAD51 and DMC1 are two RecA-like recombinases in eukaryotes. DMC1 is detected in leptotene-to-zygotene spermatocytes in mice [17]. In *Dmc1*-deficient mice, germ cells arrest in the early zygotene stage of meiosis and then undergo apoptosis [18]. Rad51 is a conserved eukaryotic protein that catalyzes the HR repair of DNA DSBs that occur during mitosis and meiosis [19]. Human and mouse RAD51 are almost identical and are highly homologous (83%) with the yeast rad51 protein [20]. RAD51 is transcribed at a high level in the thymus, spleen, testis, and ovary and at a lower level in the brain [21]. Homozygous knockout of RAD51 in mice results in early embryonic lethality and heterozygous mice appear normal and are fertile [22]. Knockdown of RAD51 by siRNAs in the seminiferous tubules of mouse testis led to reduced numbers of spermatocytes and reduced crossover formation [23]. However, the physiological role of RAD51 during spermatogenesis remains unclear.

In this study, we generated a conditional knockout mouse model to study the role of RAD51 in spermatogenesis. Conditional disruption of RAD51 in germ cells by *Vasa-Cre* led to sterility due to complete germ cell loss. We further generated a tamoxifen-inducible RAD51 knockout model by *UBC-Cre*^*ERT2*^ in adult mice. We demonstrated that RAD51 deficient male mice led to impaired DSB repair and reduced formation of crossovers during meiosis.

## Results

### RAD51 is highly expressed in the mouse testis, predominantly in germ cells

**Fig. 1:**
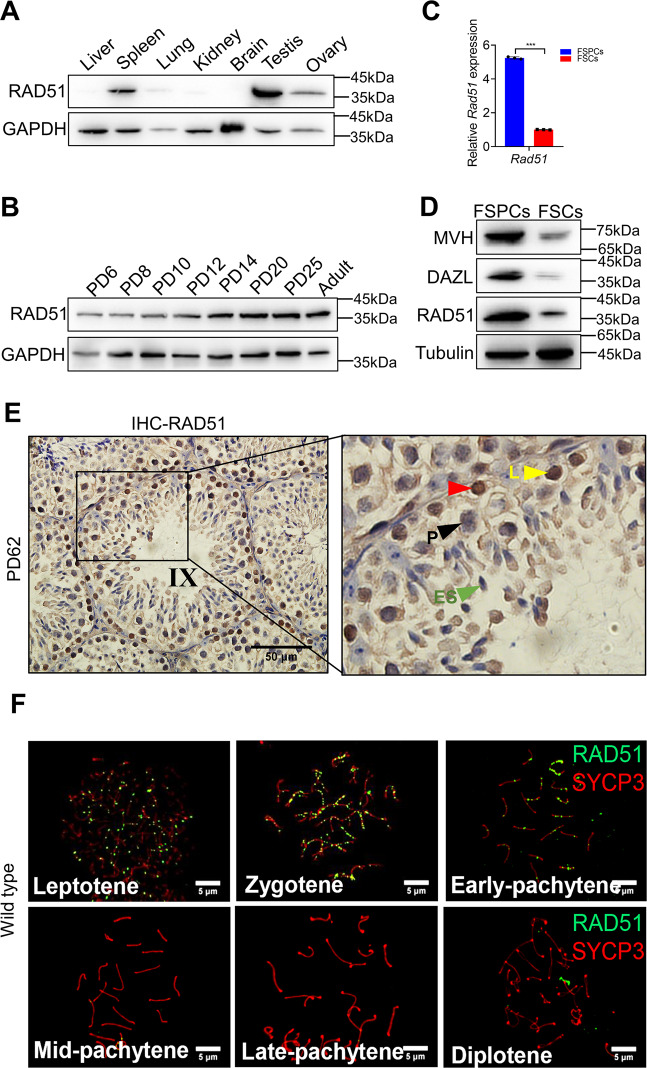
RAD51 is highly expressed in the mouse testis, predominantly in germ cells. **A** Western blotting was performed to detect RAD51 expression in different tissues of adult mice and GAPDH was used as the loading control. **B** RAD51 expression in different developmental stages of spermatogenesis was measured by Western blotting. **C**, **D** Primary isolation of spermatogenic cells and somatic cells from PD9 mouse testes. RT-qPCR (**C**) and western blotting (**D**) were conducted to test the expression of RAD51 and germ cell markers MVH and DAZL in the fraction of spermatogenic cells (FSPCs) and the fraction of somatic cells (FSCs). Data are presented as means ± S.D. (*n* = 3 biologically independent samples for RT-qPCR assay). ***p < 0.001. **E** Immunohistochemistry staining for RAD51 was analyzed in PD62 mouse testes. (red triangle: RAD51 positive cells; L: leptotene spermatocyte; P: pachytene spermatocyte; ES: elongating spermatid). Scale bar is 50 μm. **F** Immunofluorescence of RAD51 (green fluorescence) and SYCP3 (red fluorescence) on chromosome spreads in wild type. Scale bar, 5 μm.

To understand the expression and physiological role of RAD51, we measured the expression of RAD51 in different tissues of adult mice by western blot. RAD51 protein was shown to be highly expressed in the testis, spleen, and ovary (Fig. 1A). We next analyzed Rad51 expression in testes at different stages of mouse spermatogenesis using publicly available RNA-seq data [24]. We found that Rad51 had a low level of expression between E15.5 and P0 and had significantly increased levels after birth (Fig. S1A). We further performed immunoblotting to determine RAD51 level in testis tissues and found RAD51 was expressed at a moderate level from postnatal day 6 (PD6) to PD12 and then significantly increased at PD14 (Fig. 1B). Subsequently, we isolated germ cells by the differential adhesion method as described previously [25]; the enrichment efficiency was confirmed by using germ cell and somatic cell markers detected by qPCR (Fig. S1B). Then, we compared RAD51 expression between germ cells and somatic cells by qPCR and western blotting and the results revealed that RAD51 was more highly expressed in germ cells than in somatic cells (Fig. 1C, D). We also performed immunohistochemistry staining in testes and found RAD51 was highly expressed in spermatogonia and less expressed in spermatocytes (Fig. 1E, and Fig. S1C). Further, we immunostained RAD51(green) and SYCP3 (red) in meiotic chromosome spreads and found that RAD51 was expressed in leptotene and zygotene spermatocytes, and the expression diminished in mid-pachytene spermatocytes (Fig. 1F). Thus, RAD51 is expressed in mitotic spermatogonia and early meiotic prophase spermatocytes.

### Conditional disruption of RAD51 in germ cells results in sterility

**Fig. 2:**
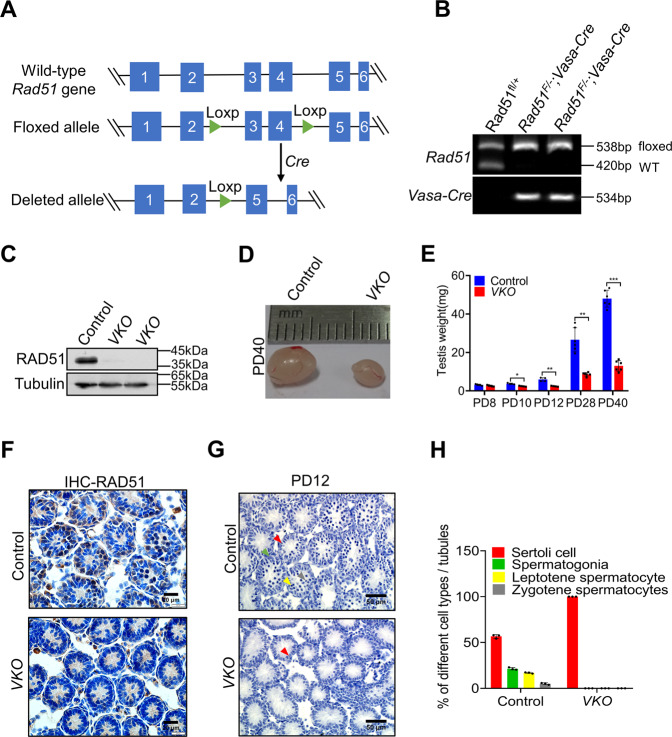
Conditional disruption of RAD51 in germ cells results in sterility. **A** A schematic diagram illustrates the generation of the Rad51 conditional knockout mice. The strategy was to insert loxp sites to flank exons 3 and 4 of the mouse *Rad51* gene. Cre recombinase mediated the removal of the floxed sequence to create a null allele. **B** Genotype identification of *Rad51* conditional knockout mice was analyzed by PCR of tail biopsy DNA. **C** The knockout efficiency was determined by western blotting in PD40 testes of *RAD51-VKO* mice compared to their littermate controls. **D** The size of testes was compared in *RAD51-VKO* males compared to their littermate controls from PD40 mice. **E** The testes weights at PD8 to PD40 were measured in *RAD51-VKO* mice and their littermate controls (*n* = 5). Data are presented as means ± S.D. *p < 0.05, **p < 0.01, ***p < 0.001. **F** Immunohistochemical staining of mouse seminiferous tubules shows the efficiency of PD8 *RAD51-VKO* mice and their littermate control. Scale bars are 20 μm. **G** Haematoxylin staining was conducted in seminiferous tubules to analyze the number and type of testicular cells in PD12 *RAD51-VKO* mice and their littermate controls. Scale bar, 50 μm. (red triangles: Sertoli cell; green triangles: spermatogonia; yellow triangles: leptotene spermatocyte; gray triangles: zygotene spermatocytes). **H** Statistics of label the different types of germ and somatic cell types to A.

To explore the function of RAD51 in spermatogenesis, we generated a conditional Rad51 null allele by flanking exons 3–4 with loxP sites (Fig. 2A). Rad51 was specifically inactivated in germ cells by crossing Rad51-floxed mice with Vasa-Cre line, which expresses CRE recombinase in male germ cells from embryonic day 15.5 [26]. The genotype of the conditional knockout mice was confirmed by PCR of tail biopsy DNA (Fig. 2B). The knockout efficiency was determined by western blotting and IHC in testes (Fig. 2C and Fig. S2A), and the results showed that RAD51 protein was absent in *Rad51-VKO* (*Rad51*^*F/-*^*; Vasa-Cre*) compared to wild-type mouse germ cells. Fertility test revealed that no pups were obtained when adult *Rad51-VKO* male mice mated with wild-type fertile female mice for at least 6 months (Table S2). We then analyzed the PD40 testis size and found that *Rad51-VKO* mice had smaller testes than their littermate controls (Fig. 2D). To obtain more information about this phenotype, we measured the testes weight at different timepoints from PD8 to PD40 and found that homozygous *Rad51-VKO* males had dramatically reduced testis weight compared to their littermate controls from PD10 to PD40, whereas no significant differences were observed in PD8 mice (Fig. 2E). Immunohistochemical staining of mouse seminiferous tubules showed that RAD51 protein decreased in PD8 testes of *Rad51-VKO* (Fig. 2F). Consistently, Hematoxylin staining demonstrated germ cell loss in PD12 and PD60 *Rad51-VKO* mice compared to their littermate controls (Fig. 2G, H and Fig. S2B). In addition, co-immunofluorescence staining of the germ cell marker GCNA (green) and RAD51 revealed that GCNA-positive cells were found both in embryonic day (E)18.5 control and *Rad51-VKO* testes (Fig. S2C), indicating germ cells were still present in *Rad51-VKO* testes at E18.5. These data indicate that RAD51 is essential for normal spermatogenesis and male fertility.

### RAD51 deletion led to Sertoli cell-only phenotype in male mice

**Fig. 3:**
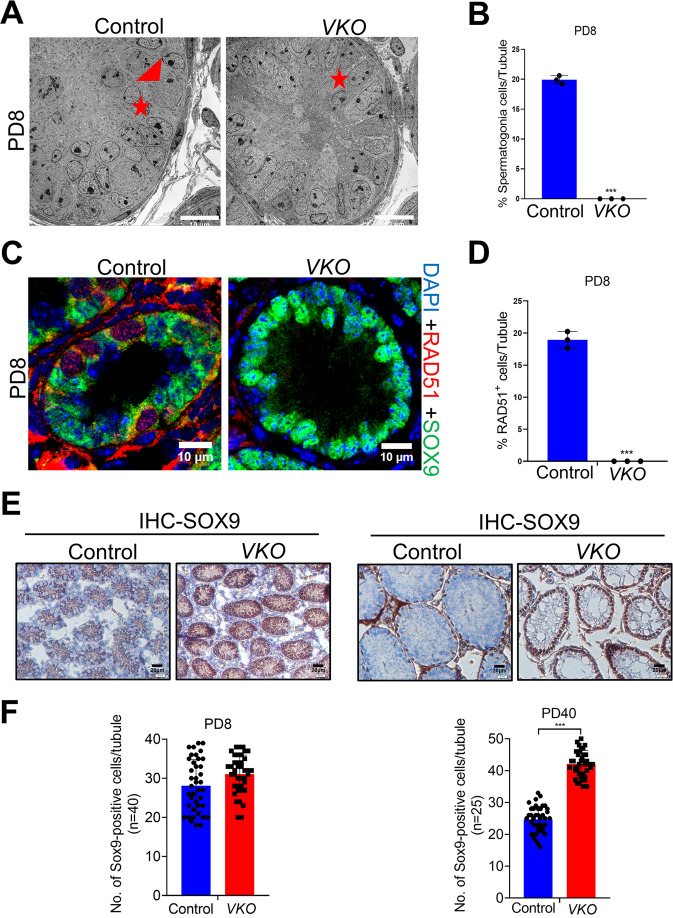
RAD51 deletion led to Sertoli cell-only syndrome in male mice. **A** Transmission electron microscopy (TEM) was performed to examine the detailed structure of seminiferous tubules at PD8 in *VKO* mice and their littermate controls (red triangles: spermatogonia; red stars: Sertoli cells). Scale bar is 10 μm. **B** Statistics of the germ cells to (**A**). Data are presented as means ± S.D. ***p < 0.001. **C** Immunofluorescence staining of the Sertoli cell marker SOX9 (green fluorescence) and RAD51 (red fluorescence) was measured in the seminiferous tubules of *RAD51-VKO* and control testes at PD8. Scale bar is 10 μm. **D** Statistics of the RAD51 positive cells to (**C**). Data are presented as means ± S.D. ***p < 0.001. **E**, **F** Immunohistochemical staining of SOX9 was analyzed in the seminiferous tubules of *VKO* and control mice at PD8 and PD40. SOX9 positive cells were counted using ImageJ. Scale bar is 20 μm. Data are presented as means ± S.D. *p < 0.05, **p < 0.01, ***p < 0.001.

We then started to investigate the underlying mechanism for the lack of germ cells in RAD51 deficient mice. Transmission electron microscopy (TEM) was used to examine the detailed structure of the seminiferous tubules at PD8. *Rad51*-deficient testes exhibited a marked depletion of germ cells in the seminiferous tubules in comparison with their littermate controls (Fig. 3A, B). Next, we performed immunofluorescence experiments to examine the early spermatogonial stem cells (SSCs) marker PLZF, the synaptonemal complex (SC) marker SYCP3, and the DNA damage marker γH2AX. No PLZF-positive cells were observed in the seminiferous tubules of *Rad51-VKO* testes at PD10 (Fig. S3A). Meanwhile, no SYCP3 or γH2AX-positive cells were observed in the seminiferous tubules of *RAD51-VKO* testes at PD8 (Fig. S3B). In addition, immunofluorescence staining of the Sertoli cell marker SOX9 revealed that the seminiferous tubules of RAD51 VKO testes contained only Sertoli cells (Fig. 3C, D and Fig. S3C, S3D). Immunohistochemical staining of SOX9 further revealed that the expression level of SOX9 and the number of Sertoli cells were increased in *RAD51VKO* mice (Fig. 3E, F and Fig. S3E–G). Thus, ablation of RAD51 results in sertoli cell-only phenotypes, indicating a pivotal role of RAD51 in spermatogenesis.

### Inducible knockout of RAD51 causes spermatogonia loss and apoptosis

**Fig. 4:**
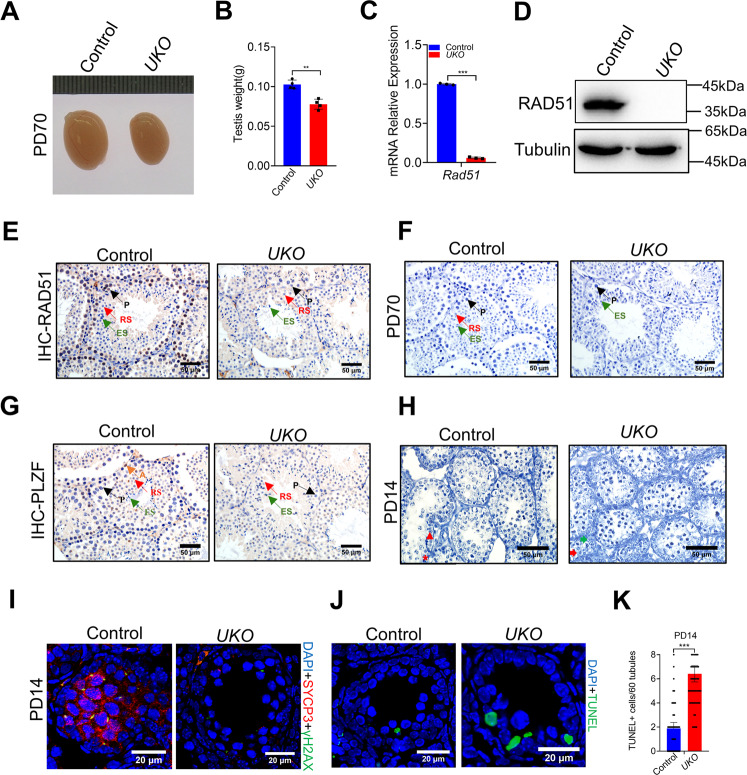
Inducible knockout of RAD51 causes spermatogonia loss and apoptosis. **A**, **B** The size (**A**) and weight (**B**) of testes were compared in mice with tamoxifen-induced knockout of *Rad51* (*Rad51*^*TAM-KO*^, *UKO*) and control mice at PD70 (*n* = 5). **C**, **D** RT-qPCR (**C**) and Western blotting (**D**) were conducted to determine the knockout efficiency of *RAD51*-*UKO* mice compared to control mice in testes. Tubulin was used as the loading control. Data are presented as means ± S.D. **p < 0.01. **E** Immunohistochemical staining of RAD51 in seminiferous tubules was used to confirm the knockout efficiency at PD70. (P: pachytene spermatocyte; RS: round spermatid; ES: elongating spermatid). Scale bar is 50 μm. **F** Haematoxylin staining was conducted in seminiferous tubules to analyze the number and type of testicular cells in *RAD51*-*UKO* and control testes at PD70. (P: pachytene spermatocyte; RS: round spermatid; ES: elongating spermatid). Scale bar is 50 μm. **G** Immunohistochemical staining of PLZF was applied to analyze in the seminiferous tubules of *RAD51*-*UKO* mice at PD70. (A: spermatogonia; P: pachytene spermatocyte; RS: round spermatid; ES: elongating spermatid). Scale bar is 50 μm. **H** Haematoxylin staining was carried out in the seminiferous tubules of *RAD51*-*UKO* and control mouse testes at PD14. (red triangle: spermatogonia; red star: pachytene spermatocytes; red arrow: type B spermatogonia; green arrow: mitotic cells). Scale bar, 50 μm. **I** Immunofluorescence staining of SYCP3 and γH2AX was applied to the seminiferous tubules of *RAD51*-*UKO* and control testes at PD14. Scale bar, 20 μm. **J** A TUNEL assay was applied to control and *RAD51*-*UKO* testes. Cells strained green are TUNEL-positive cells and DNA was stained with DAPI. Scale bar, 20 µm. **K** Statistics of a number of TUNEL-positive cells per tubule in control and *RAD51*-*UKO* testes at PD14 tubules were counted. Student’s *t*-test, error bars indicate SEM. ***P < 0.001.

Cre-ERT2 encodes a Cre fused to a mutant estrogen ligand-binding domain (ERT2) that requires tamoxifen for activity [27]. *UBC-Cre*^*ERT2*^ mice express a Cre-ERT2 fusion gene under the control of the ubiquitin C (UBC) promoter. When UBC-Cre-ERT2 mice are crossed with mice containing a *loxP*-flanked alleles of interest genes. Tamoxifen treatment could induce global knockout in the adult mouse at any age [26]. UBC-Cre ^ERT2^ mice have been used to study DDX5 in spermatogenesis [28]. To better characterize the effect of RAD51 on spermatogenesis, we crossed *UBC-Cre* ^ERT2^ mice with *Rad51*^flox/flox^ mice to generate a tamoxifen-inducible *Rad51* knockout mouse model (*Rad51*^*F/F*^*; UBC-Cre* ^*ERT2*^, *Rad51-UKO*). We then treated 8-week-olds male *UKO* (*Rad51*^*TAM-KO*^) and control mice with tamoxifen for five consecutive days and tissues were harvested at 8 days after the final injection. Subsequently, we measured the weight of the testes and found that the weight of *Rad51-UKO* testes was reduced to 80% of the weight of control testes (Fig. 4A, B). The knockout efficiency was then determined by qPCR, western blotting and IHC (Fig. 4C–E, and Fig. S4A). In addition, haematoxylin staining of seminiferous tubules showed early spermatogenic cell loss in the testes of *RAD51-UKO* mice at PD70 (Fig. 4F). In accordance with the results from *VKO* mice, immunohistochemical and immunofluorescence staining showed that PLZF-positive cells were lost in the seminiferous tubules of *RAD51-UKO* mice compared to controls at PD70 (Fig. 4G and Fig. S4B). To further investigate this phenotype, we treated PD11 *RAD51-UKO* and control mice with tamoxifen for 3 consecutive days and compared testis sections with haematoxylin staining at PD14. Unlike control testes with the normal progression of spermatocytes, in which pachytene spermatocytes could be observed, no meiotic cells were found in the testes of *RAD51-UKO* mice, only mitotic cells were observed (Fig. 4H). Furthermore, immunostaining of the meiotic markers SYCP3 and γH2AX confirmed the lack of SYCP3 and γH2AX expression in germ cells with RAD51 deletion (Fig. 4I, Fig. S4C). We speculated that the depletion of spermatogenic cells might be due to apoptosis. The TUNEL assay showed an increase in the number of apoptotic spermatocytes in the seminiferous tubules of *RAD51-UKO* mice compared to controls at PD14 (Fig. 4J, K) or PD70 (Fig. S4D, E). These data reveal that RAD51 deletion leads to early spermatogenic cell loss and apoptosis.

### Inducible knockout of Rad51 leads to meiosis defects

**Fig. 5:**
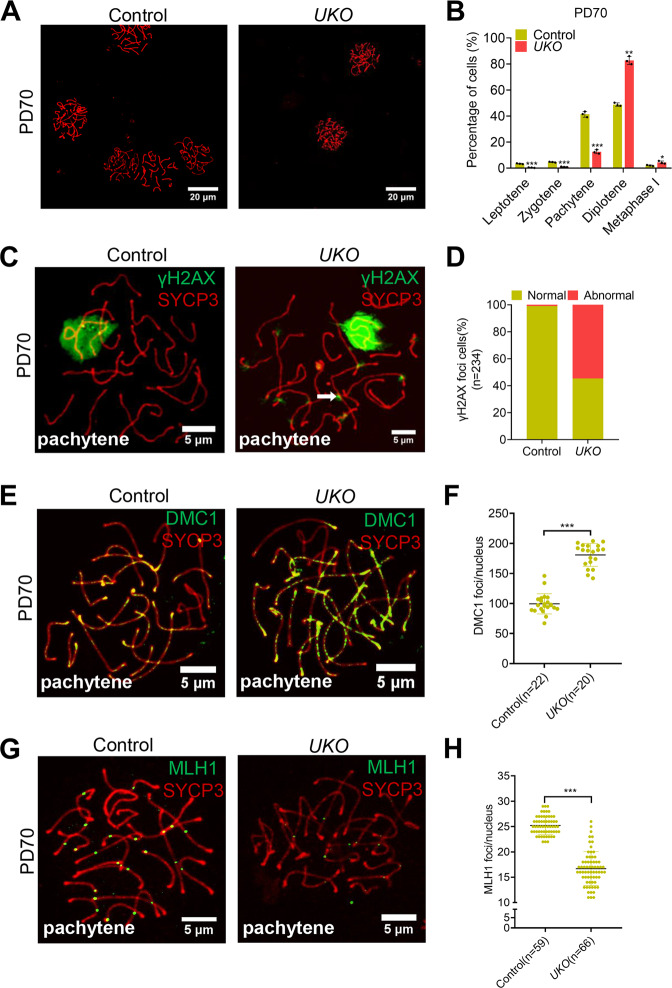
Inducible knockout of RAD51 exhibits meiosis defects in male mice. **A** Immunofluorescence of SYCP3 expression shown on chromosome spreads from control and *RAD51*-*UKO* testes at PD70. Scale bar, 20 μm. **B** Statistics of each meiotic period corresponding to (**A**). **C**, **D** Immunofluorescence of SYCP3 and γH2AX on chromosome spreads from control and *RAD51*-*UKO* testes at PD70 was shown and counted. Scale bar, 5 μm. **E**, **F** Immunofluorescence of SYCP3 and DMC1 on chromosome spreads in control and *RAD51*-*UKO* testes at PD70 was shown and counted. Scale bar, 5 μm. **G**, **H** Immunofluorescence of SYCP3 and MLH1 on chromosome spreads from control and *RAD51*-*UKO* testes at PD70 was shown and quantified. Scale bar, 5 μm. Data are presented as means ± S.D.*p < 0.05, **p < 0.01, ***p < 0.001.

While RAD51 is believed to be essential for both mitotic and meiotic recombination. However, the role of RAD51 in meiosis remains poorly characterized in mice due to the embryonic lethality of the RAD51 homozygous knockout mice. We sought to investigate the functions of RAD51 in male meiosis using a tamoxifen-inducible *Rad51* knockout mouse model treated with tamoxifen as described above. We immunostained the testis sections for the meiotic marker SYCP3 and performed a quantitative analysis of spermatocytes at different meiotic stages in *RAD51-UKO* and control mice. Interestingly, compared to the profile in control mice, we observed a significant increase in the proportion of diplotene spermatocytes (from 48.83% to 82.74%) and a decrease in the proportion of pachytene spermatocytes (from 41.37 to 12.57%) in *RAD51-UKO* mice (Fig. 5A, B and Fig. S5A). To further investigate the role of RAD51 in the repair of meiotic DSBs, we performed immunostaining of γH2AX, a marker of meiotic DSBs, on spread spermatocytes. We observed the accumulation of γH2AX foci on the axes of autosomal chromosomes in pachytene spermatocytes in *RAD51-UKO* mice (Fig. 5C, D and Fig. S5B). Similarly, we observed a significant increase of DMC1 foci in the pachytene stage in *RAD51-UKO* mice compared to control mice (Fig. 5E, F and Fig. S5C). These data indicate that depletion of RAD51 results in an increased incidence of unrepaired DNA breaks. Moreover, we immunostained MLH1 to evaluate crossover maturation in the pachytene stage and found a significant decrease in MLH1 foci in *RAD51-UKO* mice compared to control mice (Fig. 5G, H and Fig. S5D). These data reveal that RAD51 is critical for DSB repair and meiotic progression.

## Discussion

Rad51 mediates the HR repair both in mitosis and meiosis [29]. However, because of the early embryonic lethality of constitutive RAD51 knockout mice, the importance of RAD51 in spermatogenesis has not been fully elucidated. In this study, we explore the physiological role of RAD51 in spermatogenesis using a conditional knockout mouse model for the first time. We found that RAD51 was highly expressed in germ cells of mouse testis. Conditional disruption of RAD51 in germ cells with Vasa-Cre results in SSCs loss and Sertoli cell-only phenotype. In a tamoxifen-inducible Rad51 knockout mouse model, we observed that RAD51 deletion led to early spermatogenic cells loss and apoptosis. Moreover, we demonstrate that RAD51 ablation results in defective DSB repair and crossover formation of male meiosis.

Rad51 mutations in mice cause early embryo lethality, indicating the essential function of RAD51 in mitotic development [22]. In budding yeast, rad51 mutants show reduced spore formation and viability [30]. Loss of the RAD51 function in mice results in a defect in mitosis that is accompanied by chromosome fragmentation [31]. Disruption of the RAD51 gene in the chicken B-cell line DT40 led to G2/M phase arrest, and eventually dying [32]. However, loss-of-function mutant in the Arabidopsis homolog of RAD51 has no detectable abnormality in mitosis. Our findings showed that RAD51 deletion led to early spermatogenic cells loss and apoptosis, supporting the essential role of RAD51 in mitosis. A previous study reported that RAD51 contributes to G2/M transition in mouse embryonic stem cells [33]. RAD51 is responsible for recombinational repair of DNA damage during mitosis [34]. RAD51 also plays non-repair functions, which stimulates replication forks reversal and stabilizes stalled forks [35]. RAD51 promotes mitotic DNA synthesis and successful chromosome segregation in mitosis [36].

In meiosis, we found that inducible knockout of RAD51 at PD14 (three days post tamoxifen treatment), germ cells failed to enter meiosis as evidenced by histological analysis and the lack of SYCP3 and γH2AX expression (Fig. 4I). Nevertheless, a previous study shows microinjection of siRNAs into seminiferous tubules of mouse testis results in no significant reduction of EdU positive cells in the RAD51 knockdown tubules [23]. The discrepancy may be explained by the incomplete knockdown of RAD51 in mouse testis since heterozygous RAD51 knockout mice are viable and fertile [37]. Both RAD51 and DMC1 are essential for HR during meiosis and they might have distinct and overlapping roles in recombination. In yeast, Rad51 foci form normally in Dmc1 mutant strain whereas the formation of Dmc1 foci is greatly reduced in Rad51 mutant cells [38]. In budding yeast, Dmc1-deficient strain exhibits defects in meiotic recombination and SC formation and cells arrested at the pachytene stage [39]. Similar but not the same, spermatocytes of DMC1 knockout mice are arrested at the early zygotene stage, with the failure of homologous chromosomes to undergo synapsis [18]. DMC1 and RAD51 have distinct spatial localization on ssDNA [14]. In yeast, Rad51 is an accessory factor for the strand exchange activity of Dmc1 during meiosis [19]. However, our study revealed that the inducible knockout of RAD51 led to a significant decrease in the proportion of zygotene and pachytene spermatocytes relative to control mice (Fig. 5A, B). In addition, we found that DMC1 foci were increased in the pachytene stage in RAD51 deficient spermatocytes (Fig. 5E, F), suggesting that RAD51 and DMC1 have concerted functions during meiotic HR in male mice. Our study demonstrates that RAD51 is essential for meiotic recombination in mice. Further studies are needed to elucidate the role of RAD51 and DMC1 in mammalian meiosis.

In summary, we demonstrated that RAD51 is essential for spermatogonial mitosis as well as meiotic recombination in mice. Inactivation of *Rad51* causes spermatogonia loss and Sertoli cell-only phenotype. Inducible knockout of *Rad51* leads to leads to meiosis defects, with a significant decrease of zygotene and pachytene spermatocytes. Thus, RAD51 is indispensable for the survival of spermatogonia as well as meiotic progression in mice.

## Materials and methods

### Mice

*Rad51-*floxed line was generated by flanking exon 3 and 4 of *Rad51* with *loxP* sites (Constructed at Nanjing Model Organism Center). The resulting founder male mice were mated to WT C57BL/6 J (B6) female mice to obtain heterozygous *Rad51*-floxed mice. Progeny was screened by PCR for germ line transmission of the targeted alleles. All mice described above were maintained in C57BL/6J (B6) background. *Rad51-* floxed mice (*Rad51*^flox/flox^) were then crossed to *Vasa-Cre* mouse line (Jackson laboratory) [26]. All animal experiments were conducted in accordance with the guidelines in the Animal Care and Use Committee at Shandong University. To generate tamoxifen-inducible *Rad51* knockout mice, *Rad51*^flox/flox^*-UBC-Cre*^*ERT2*^ mice (which were obtained from the Jackson Laboratory) were subjected to tamoxifen treatment. Tamoxifen (Sigma, T5648) was dissolved in corn oil at a concentration of 20 mg/ml and injected into the abdominal cavity of 8-week-old male mice at a dose of 4 mg/30 g body weight for five consecutive days and tissues were harvested at 8 days after the final injection [28, 40]. The tamoxifen-inducible *Rad51* knockout mice were referred to as *Rad51-UKO* mice. All the primers for PCR genotyping were listed in Supplementary information, Table S1.

### Enrichment of spermatogenic cells

The spermatogenic cells and somatic cells were enriched using a two-step enzymatic digestion process followed by a differential adhesion method as previously described with some modifications [41]. Briefly, after the tunica albuginea was disrupted, testes from postnatal day (PD) nine wild-type mice were transferred into 120 U/ml collagenase type I (Gibco, 17100-017) and incubated at 37 °C for 20 min with gentle shaking every 3–5 min to accelerate testis dissociation. Then, the cell suspension was centrifuged at 1000 rpm. for 5 min, and the pellet was digested with 0.25% trypsin-EDTA (Gibco, 25200-072) at 37 °C for 10 min to dissociate the seminiferous tubules into single cells. The suspension was neutralized with 5 ml DMEM supplemented with 10% FBS and centrifuged at 1000 rpm. for 5 min. The cell pellet was suspended in 8 ml of DMEM medium and seeded in a 10 cm culture dish. After 2–3 h of incubation at 37 °C, the floating and weakly adhering cells were transferred to a new 10 cm dish. The fraction of spermatogenic cells (FSPCs) comprised the floating and weakly adhering cells. The attached cells on the bottom of the dish were collected as the fraction of somatic cells (FSCs). The efficiency of separation was examined using RT-PCR and western blotting analysis.

### Histological analysis, immunostaining, and imaging

Testes from control and *RAD51-VKO/UKO* male mice were isolated and fixed in 4% paraformaldehyde (PFA) overnight at 4 °C for immunostaining. The samples were dehydrated stepwise through an ethanol series (25, 50, 75, 85, 95, and 100% ethanol), embedded in paraffin and sectioned (4 μm). After dewaxing and hydration, the sections were stained with haematoxylin and imaged with an Olympus microscope. For immunostaining, after dewaxing and hydration, the section was placed in boiling EDTA and heated for 15 min for antigen retrieval. After washing with PBS three times, the sections were permeabilized with 0.2% Triton X-100 for 15 min, washed with PBS three times, blocked with 5% BSA for 1 h at room temperature and incubated overnight at 4 °C with primary antibodies diluted in western primary antibody diluent (Beyotime, P0023A). The next day, following three washes with PBS, secondary antibodies were added to the sections and incubated for 1 h at room temperature. The sections were then washed in PBS three times, washed with water several times, and incubated with DAPI (Abcam, ab104139) and sealed with nail polish. The primary antibodies used were as follows: rabbit anti-RAD51 polyclonal antibody (#PA5-27195, Thermo Fisher Scientific, 1:200); mouse anti-PLZF monoclonal antibody (sc-28319, Santa Cruz, 1:200); mouse anti-phospho-histone H2AX (Ser139/Tyr142) antibody (#05-636, Millipore, 1:300); mouse anti-SCP3 monoclonal antibody (sc-74569, Santa Cruz, 1:200); rabbit anti-SCP3 monoclonal antibody (sc-33195, Santa Cruz, 1:200); and rabbit anti-SOX9 monoclonal antibody (ab185966, Abcam, 1:250).

### RNA extraction and real-time qPCR

Total RNA was extracted from whole testes using TRIzol reagent (Invitrogen, 15596-026) following the manufacturer’s instructions. After removing the residual genomic DNA, 1 μg of total RNA was reverse-transcribed into cDNA using the HiScript III RT SuperMix for qPCR (+gDNA wiper) (Vazyme, R223-01) according to the manufacturer’s protocol. Real-time RT-PCR was performed using ChamQ SYBR Color qPCR Master Mix (Vazyme, Q411-02) on a Quant Studio 3 (Thermo Fisher). The primers for qPCR assay are listed in Table S1.

### Western blotting

Protein samples were prepared using cell lysis buffer for western blotting and IP without inhibitors mixed with a protease inhibitor cocktail (Beyotime, P011) and quantified using a BCA reagent kit (Beyotime, P0012-1). Equal amounts of total protein were separated in a 12% SDS–PAGE gel and transferred onto PVDF membranes. After blocking with 5% nonfat milk for 1 h at room temperature, the membranes were incubated with diluted primary antibodies at 4 °C overnight. After three washes with TBST, the membranes were incubated with horseradish peroxidase-conjugated secondary antibodies (1:10,000, Jackson ImmunoResearch) at room temperature for 1 h. The signals were developed with Pierce ECL Substrate (Thermo Fisher Scientific, #34080). The primary antibodies were as follows: rabbit anti-RAD51 polyclonal antibody (#PA5-27195, Thermo Fisher Scientific, 1:10,000); rabbit anti-DAZL polyclonal antibody (ab34139, Abcam, 1:5000); rabbit anti-DDX4/MVH polyclonal antibody (ab13840, Abcam, 1:5000); mouse anti-GAPDH monoclonal antibody (60004-1-Ig, Proteintech, 1:10,000); and mouse anti-tubulin monoclonal antibody (66031-1-Ig, Proteintech, 1:10,000).

### Statistical analysis

The results of all quantitative experiments were based on at least three independent biological samples. All data are presented as means ± SEM. The statistical significance of the differences between the mean values for the different genotypes was measured by Student’s two-tailed *t*-test with a paired *t*-test. The data were considered significant when the *p* value was <0.05.

## Supplementary information

Supplementary Figure 1

Supplementary Figure 2

Supplementary Figure 3

Supplementary Figure 4

Supplementary Figure 5

Supplementary Figure Legends

Supplementary Tables

Original data files

## Data Availability

The data that support the findings of this study are available from the corresponding author upon reasonable request.
