# Supplementary figures and images for: RAD51 is essential for spermatogenesis and male fertility in mice
Source: Cell Death Discov. 2022 Mar 15;8:118. doi: 10.1038/s41420-022-00921-w (PMC8924220; doi:10.1038/s41420-022-00921-w)

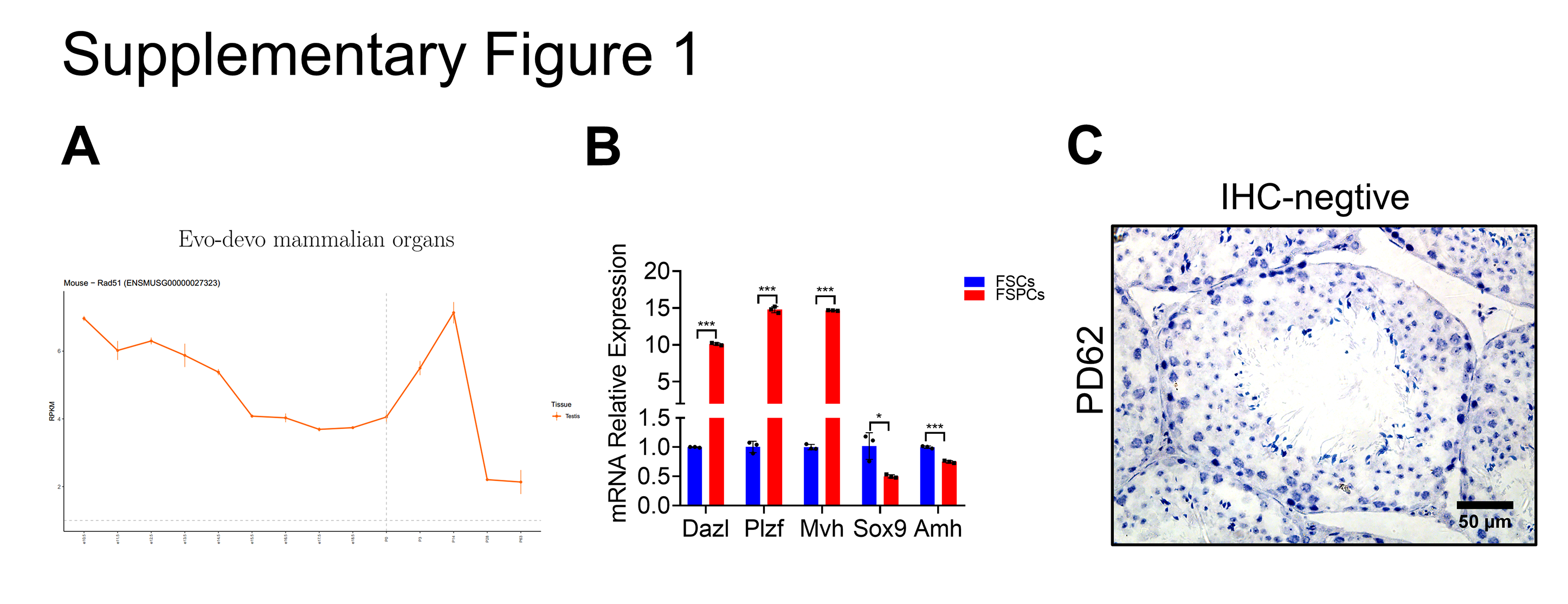

Supplement: Supplementary file 1 — Supplementary Figure 1 [file 41420_2022_921_MOESM1_ESM.tif]

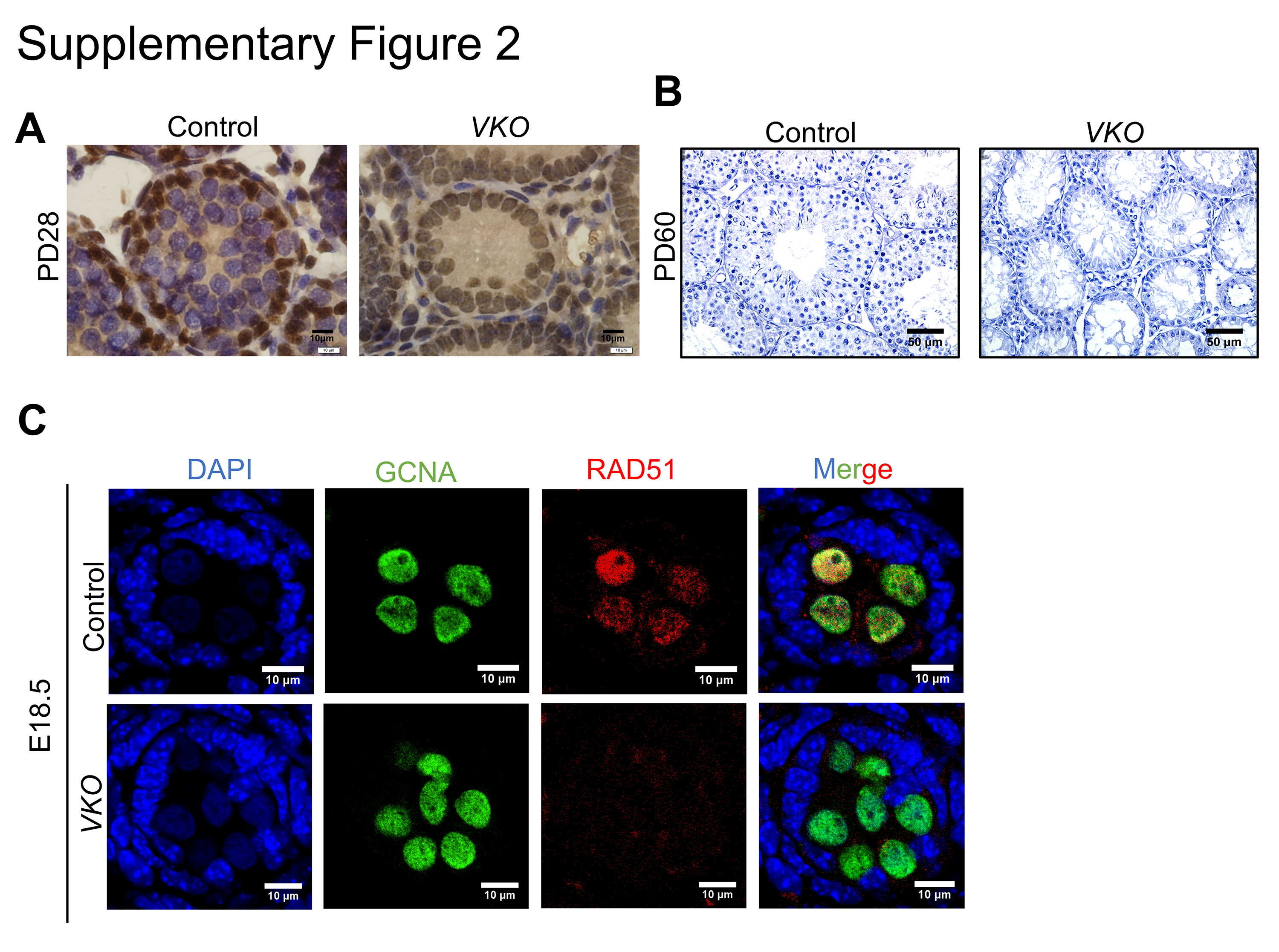

Supplement: Supplementary file 2 — Supplementary Figure 2 [file 41420_2022_921_MOESM2_ESM.tif]

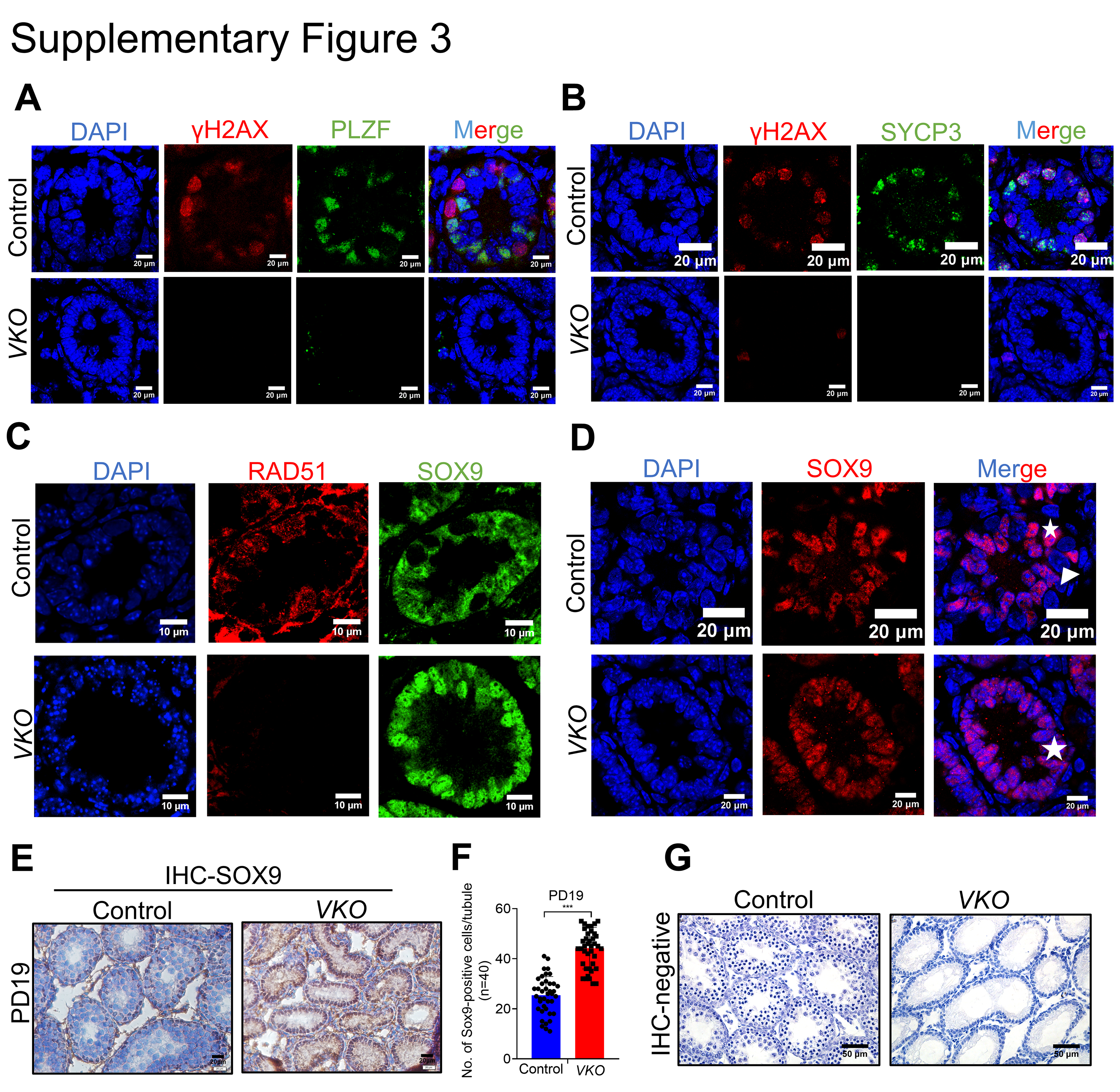

Supplement: Supplementary file 3 — Supplementary Figure 3 [file 41420_2022_921_MOESM3_ESM.tif]

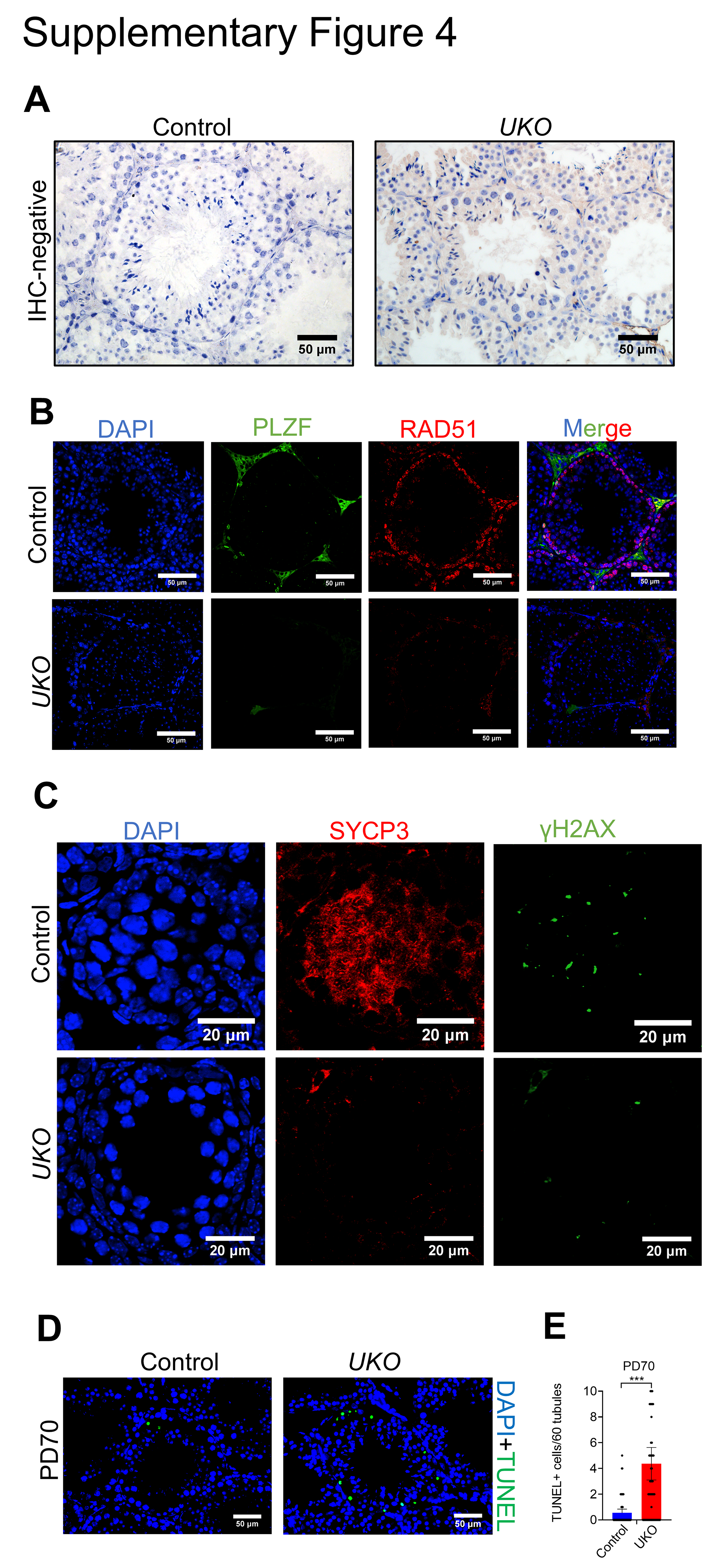

Supplement: Supplementary file 4 — Supplementary Figure 4 [file 41420_2022_921_MOESM4_ESM.tif]

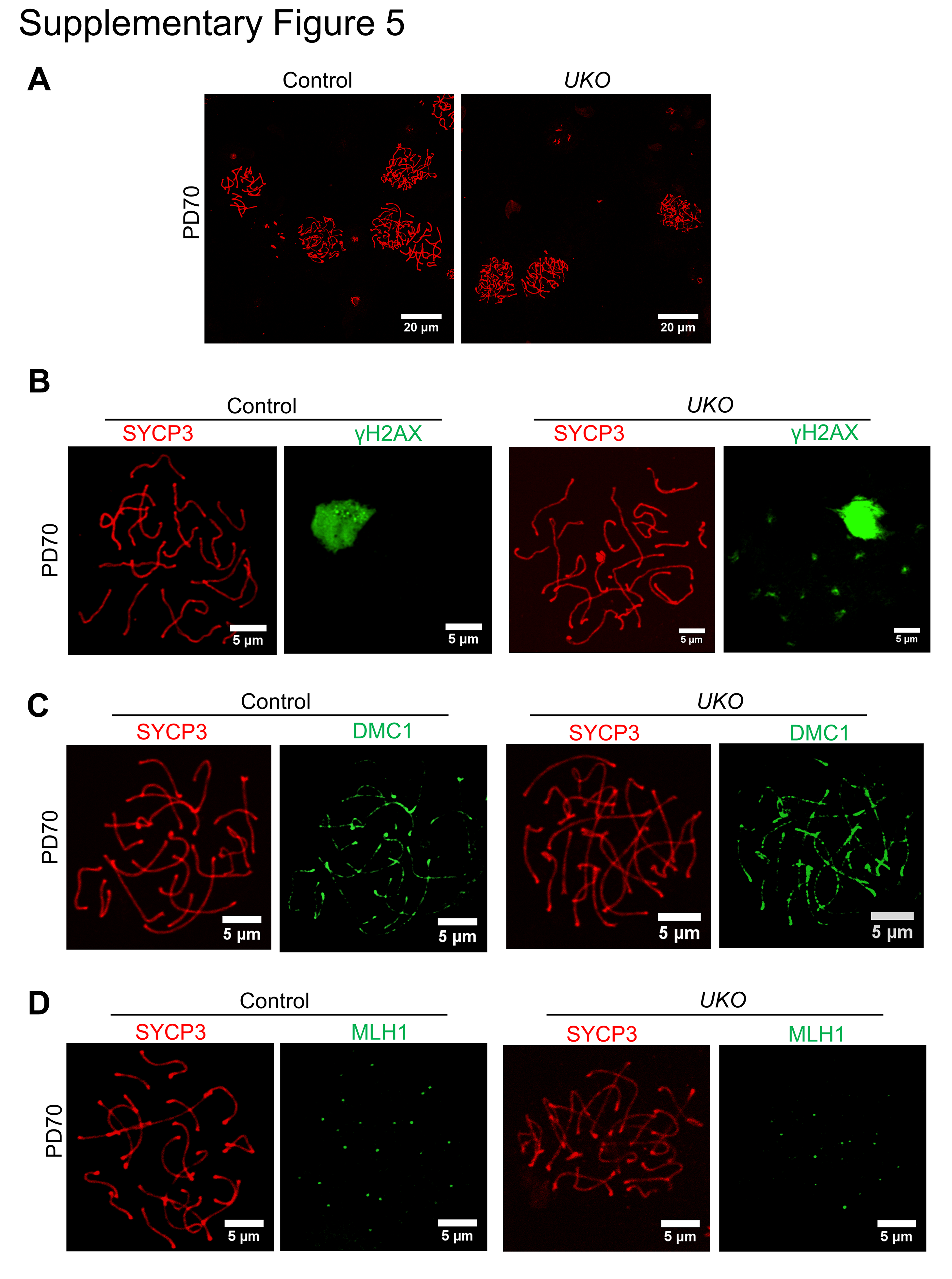

Supplement: Supplementary file 5 — Supplementary Figure 5 [file 41420_2022_921_MOESM5_ESM.tif]
