# Supplementary material for: RAD51 is essential for spermatogenesis and male fertility in mice
Source: Cell Death Discov. 2022 Mar 15;8:118. doi: 10.1038/s41420-022-00921-w (PMC8924220; doi:10.1038/s41420-022-00921-w)
Supplement: Supplementary file 6 — Supplementary Figure Legends [file 41420_2022_921_MOESM6_ESM.docx]

**Supplementary Figure Legends**

**Fig. S1 Expression of RAD51 in germ cell mitosis and meiosis. A** The Rad51 expression in testes at different stages of mouse spermatogenesis using evo-devo mammalian organs. **B** RT-qPCR analysis of RAD51 expression in the fraction of spermatogenic cells (FSPCs) and the fraction of somatic cells (FSCs) enriched from PD9 male testes. Germ cell markers (Dazl, Plzf and Mvh) and somatic cell markers (Sox9 and Amh) were used as the indicator of the enrichment efficiency. **C** The negative of immunohistochemistry staining for RAD51 was analyzed in PD62 mouse testes. Scale bars is 50 μm.

**Fig. S2 RAD51 expression was measured in testes of *Rad51****-****VKO* mice. A** Immunohistochemistry staining against RAD51 in the seminiferous tubules of *Rad51-VKO* and control testes at PD28. Scale bar, 10μm. Data are presented as means ± S.D. **B** Haematoxylin staining of seminiferous tubules in PD60 *Rad51-VKO* mice and their littermate control. Scale bars are 50 μm. **C** Immunofluorescence staining of the germ cell marker GCNA (green fluorescence) and RAD51 (red fluorescence) was measured in the seminiferous tubules of *VKO* and control testes at E18.5. Scale bar is 10μm.

**Fig. S3** **RAD51 deletion led to** **sertoli cell-only syndrome in male mice. A** Immunofluorescence staining of the stem cell marker PLZF (green fluorescence) and meiotic marker γH2AX (red fluorescence) expression was analyzed in the seminiferous tubules of *Rad51-VKO* and control testes at PD10. Scale bar is 20 μm. **B** Immunofluorescence staining of the meiotic marker SYCP3 (green fluorescence) and γH2AX (red fluorescence) was measured in the seminiferous tubules of *Rad51-VKO* and control testes at PD8. Scale bar is 20 μm. **C** Immunofluorescence staining of SOX9 (green fluorescence) and RAD51 (red fluorescence) in the seminiferous tubules of *Rad51-VKO* and control testes at PD8. Scale bar, 10μm. **D** Immunofluorescence staining of the Sertoli cell marker SOX9 (red fluorescence) was measured in the seminiferous tubules of *Rad51-VKO* and control testes at PD8. Scale bar is 20μm. (White triangles: spermatogonia; white stars: Sertoli cells) Data are presented as means ± S.D. **E-F** Immunohistochemical staining of SOX9 was analyzed in the seminiferous tubules of *VKO* and control mice at PD19. SOX9 positive cells were counted using ImageJ. Scale bar is 20 μm. Data are presented as means ± S.D. *p < 0.05, **p < 0.01, ***p < 0.001. **G** The negative of immunohistochemistry staining for SOX9 was analyzed in PD40 mouse testes. Scale bars is 50 μm.

**Fig. S4** **Inducible Knockout of RAD51 causes spermatogonia loss and apoptosis. A** The negative of immunohistochemistry staining for *Rad51-UKO* testes but not in control mice at PD70. Scale bars is 50 μm. **B** Immunofluorescence staining of PLZF (green fluorescence) and RAD51 (red fluorescence) in the seminiferous tubules of *Rad51-UKO* and control testes at PD70. Scale bar, 20μm. **C** Immunofluorescence staining of SYCP3 (green fluorescence) and γH2AX (red fluorescence) in the seminiferous tubules of *Rad51-UKO* and control testes at PD14. SYCP3 and γH2AX fluorescent staining was not observed in *Rad51-UKO* mice. Scale bar, 20μm. **D** TUNEL-positive cells were observed in seminiferous tubules of *Rad51-UKO* testes but not in control mice at PD70. The DNA was stained with DAPI. Scale bar, 20 µm. Data are presented as means ± S.D. **E** Statistics of number of TUNEL-positive cells per tubule in control and *Rad51-UKO* testes at PD70 tubules were counted. Student’s t-test, error bars indicate SEM. ***P < 0.001.

**Fig. S5 Inducible knockout of RAD51 exhibits meiosis defects in male mice. A** Immunofluorescence of SYCP3 on chromosome spread in *Rad51-UKO* and control testes at PD70. Scale bar, 20μm. **B** Immunofluorescence of SYCP3 (Red) and γH2AX (Green) on chromosome spreads in control and *Rad51-UKO* testes at PD70. Scale bar, 5μm. **C** Immunofluorescence of SYCP3 (Red) and DMC1 (Green) on chromosome spreads in control and *Rad51-UKO* testes at PD70. Scale bar, 5μm. **D** Immunofluorescence of SYCP3 (Red) and MLH1 (Green) on chromosome spreads in control and *Rad51-UKO* testes at PD70. Scale bar, 5μm. Data are presented as means ± S.D.
