# Supplementary material for: RAD51 is essential for spermatogenesis and male fertility in mice
Source: Cell Death Discov. 2022 Mar 15;8:118. doi: 10.1038/s41420-022-00921-w (PMC8924220; doi:10.1038/s41420-022-00921-w)
Supplement: Supplementary file 7 — Supplementary Tables [file 41420_2022_921_MOESM7_ESM.docx]

Table S1. Primer sequence used for genotyping assay

| Gene | Forward (5’-3’) | Reverse (5’-3’) |
| --- | --- | --- |
| *Rad51* | AAGCCAGAGAATGCAAGTATCTC | CACCTCAGAGAAGTGTTCGAAGA |

Primer sequence used for qPCR assay

| Gene | | | Forward (5’-3’) | Reverse (5’-3’) |
| --- | --- | --- | --- | --- |
| *Rad51* | | GCGCCGGTCAGAGATCATAC | | TGGCATGTAACAGCCAACGTA |
| *Gapdh* | CCCCAATGTGTCCGTCGTG | | | TGCCTGCTTCACCACCTTCT |
| *Dazl* | GGATGAAACCGAAATCAGGA | | | ATAGCCCTTCGACACACCAG |
| *Plzf* | CTGGGACTTTGTGCGATGTG | | | CGGTGGAAGAGGATCTCAAACA |
| *Amh* | CCACACCTCTCTCCACTGGTA | | | GGCACAAAGGTTCAGGGGG |
| *Sox9* | CGGAACAGACTCACATCTCTCC | | | GCTTGCACGTCGGTTTTGG |
| *Mvh* | CTAGGAAGACCAAATAGTGAATCTGAC | | | TCCAGAACCTGTTACTACTTCTTCATT |

Table S2. Fertility test of Rad51 VKO mice

| Genotype | | No. of male mice | No. of litters | No. of pups | No. of pups per litter |
| --- | --- | --- | --- | --- | --- |
| Male  (8-week-old) | Female  (8-week-old) |  |  |  |  |
| Control | Wt | 5 | 41 | 275 | 6.71 |
| VKO | Wt | 5 | 0 | 0 | 0 |
