# Supplementary material for: RAD51 is essential for spermatogenesis and male fertility in mice
Source: Cell Death Discov. 2022 Mar 15;8:118. doi: 10.1038/s41420-022-00921-w (PMC8924220; doi:10.1038/s41420-022-00921-w)
Supplement: Supplementary file 8 — Original data files [file 41420_2022_921_MOESM8_ESM.docx]

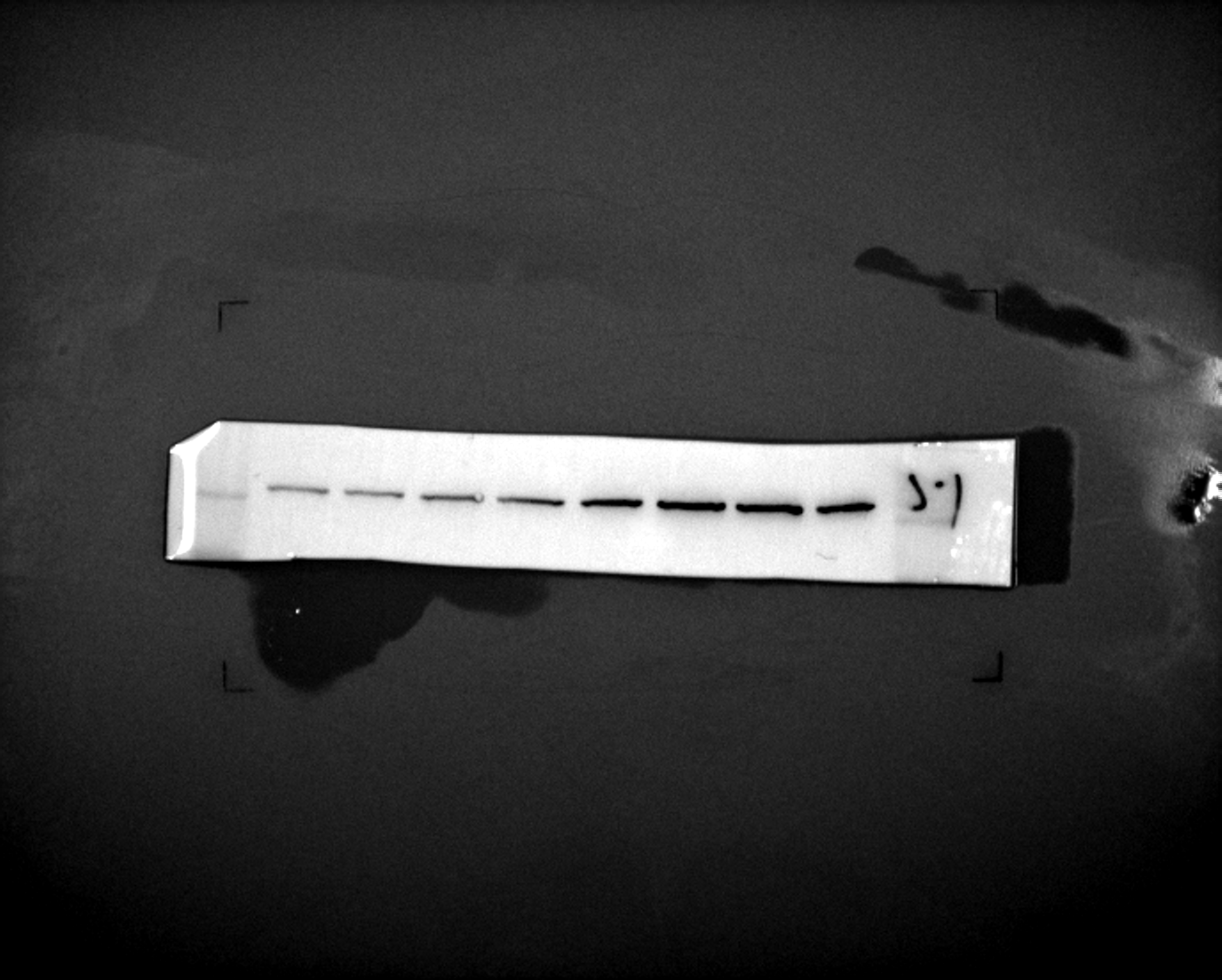

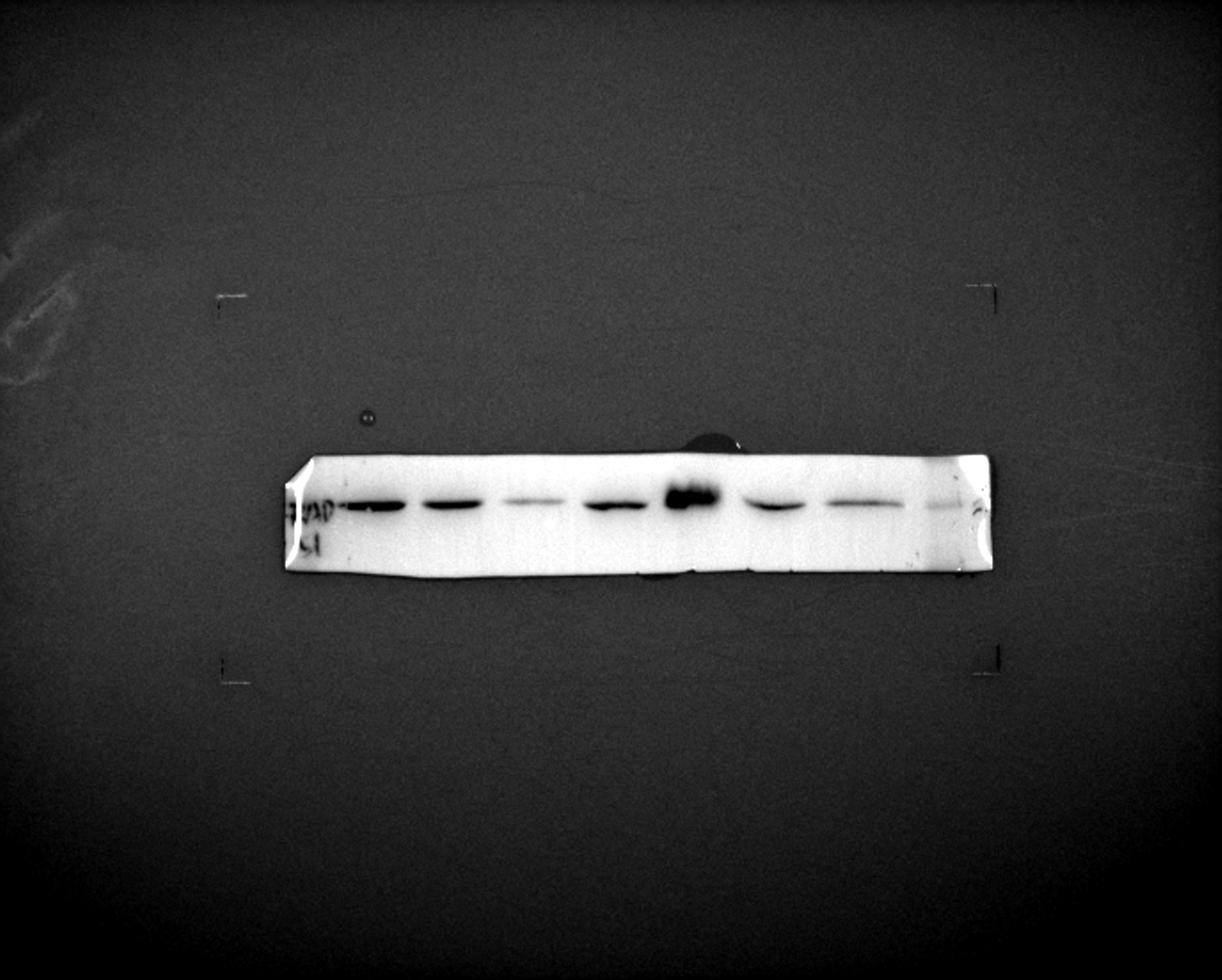

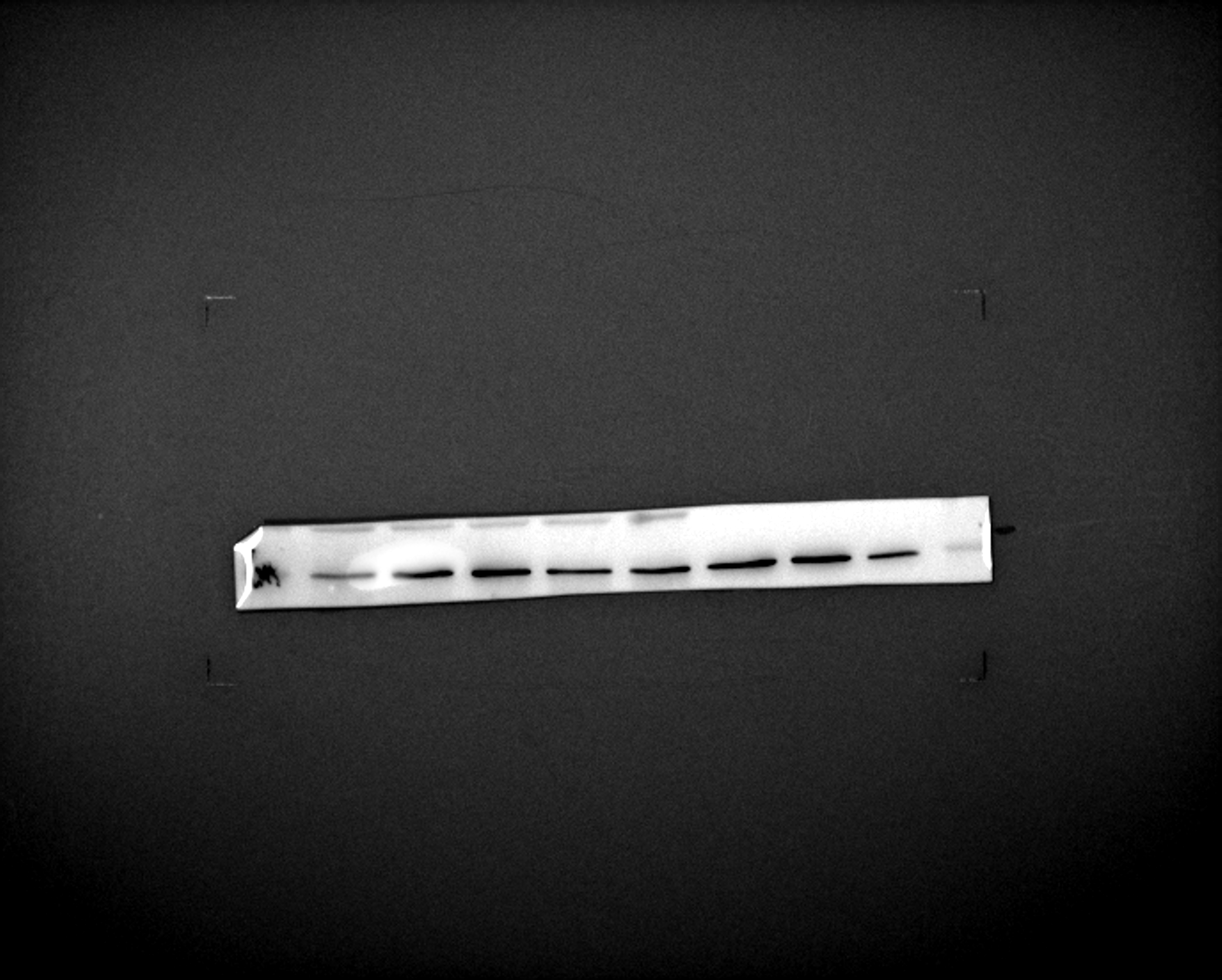

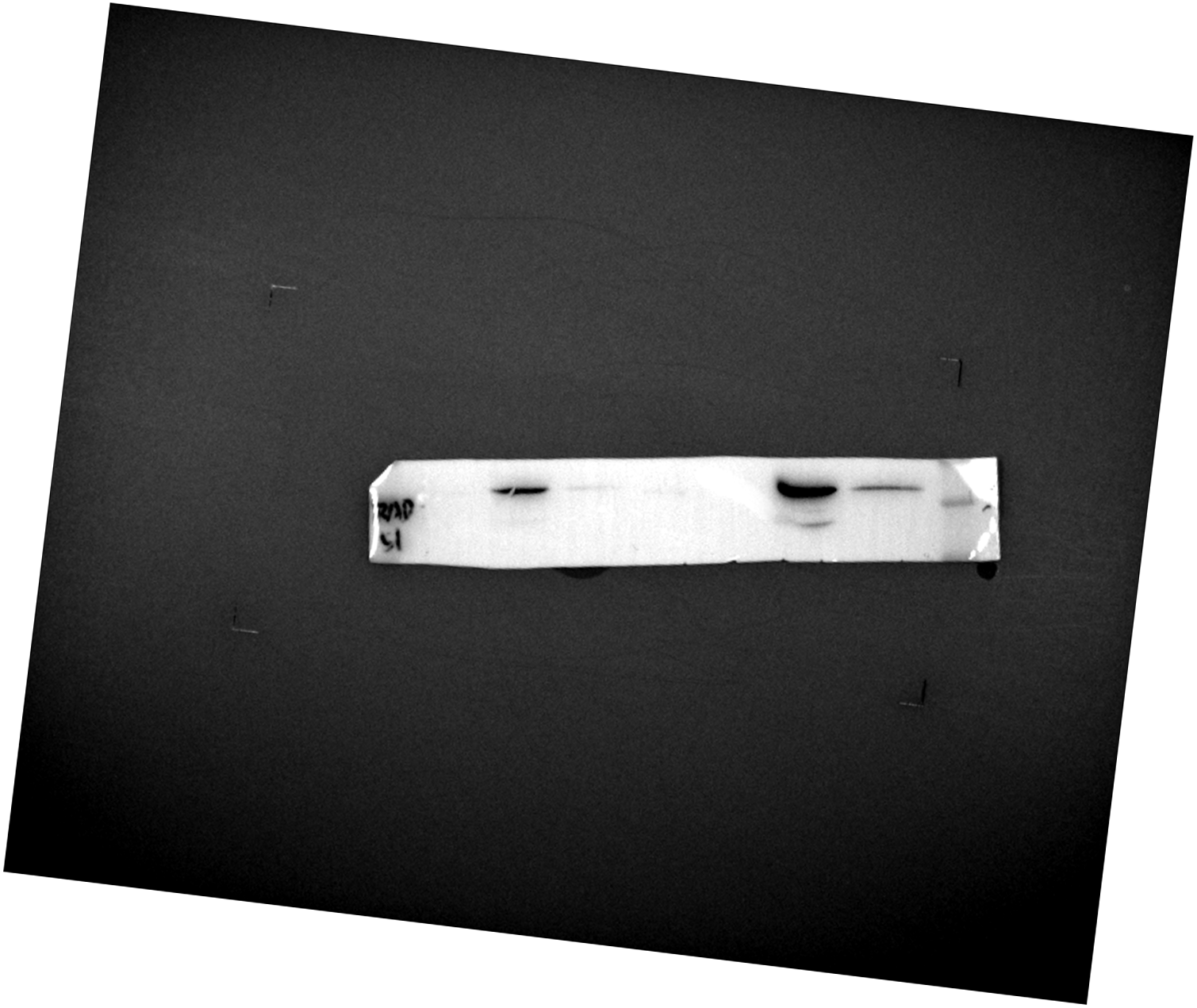

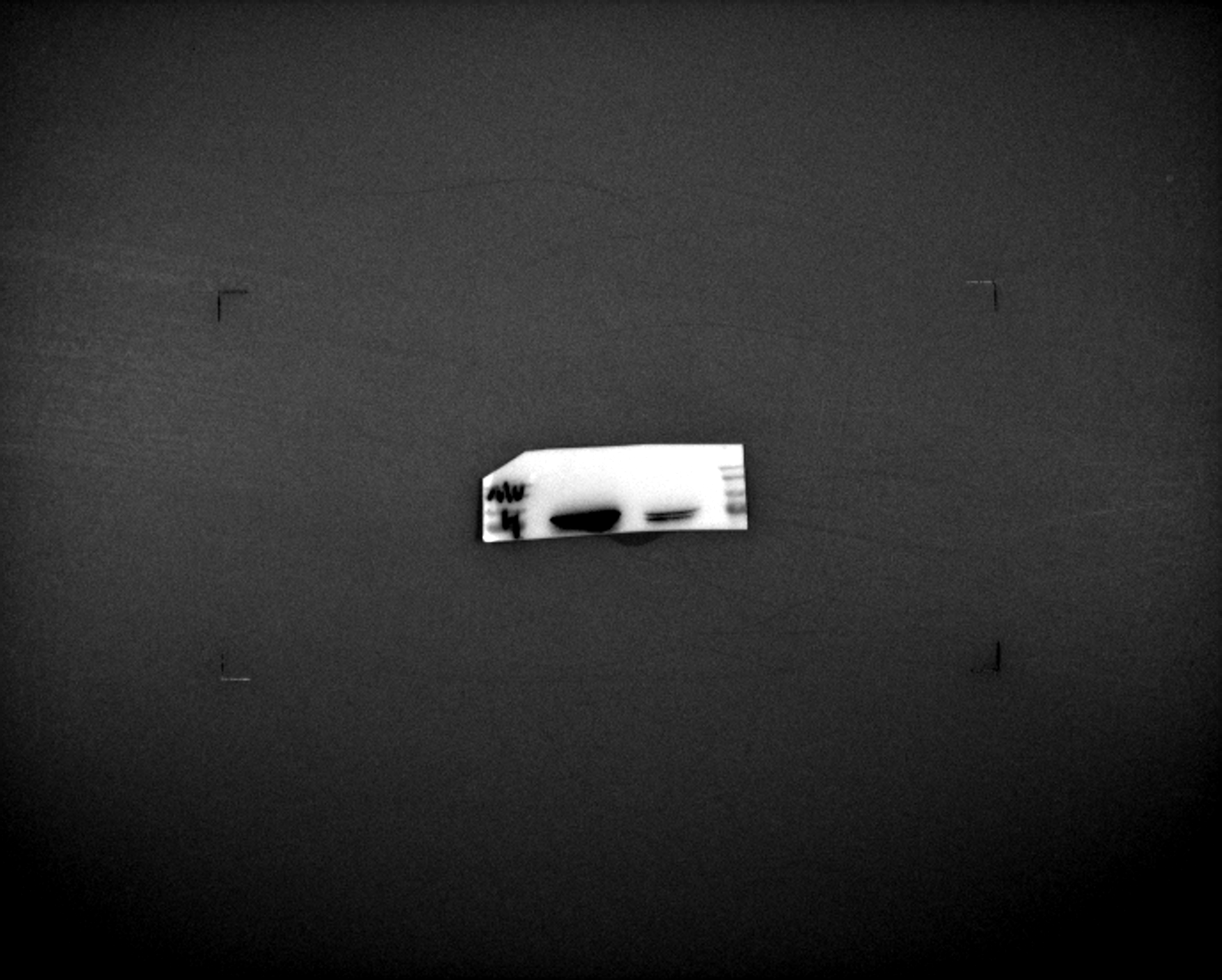

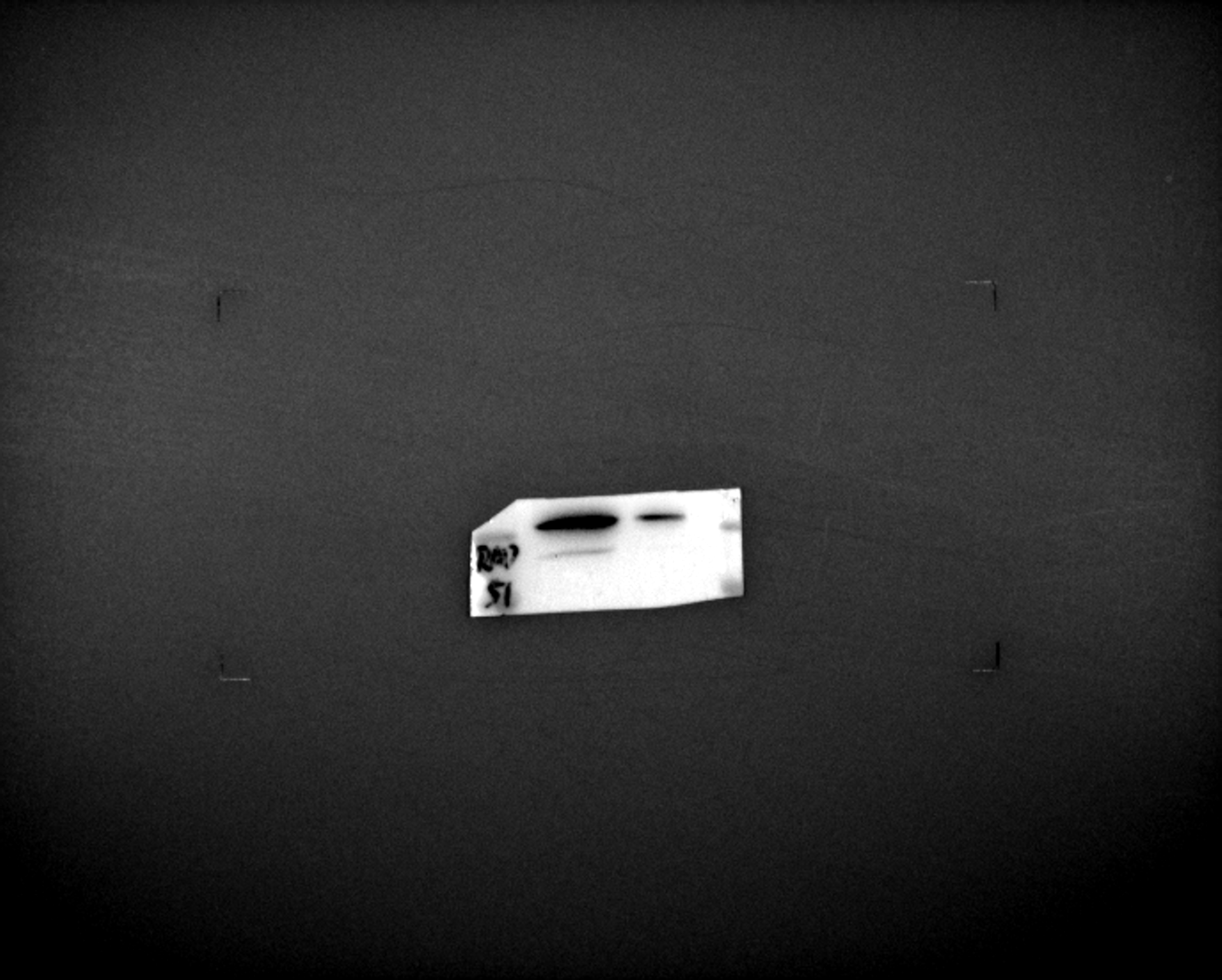

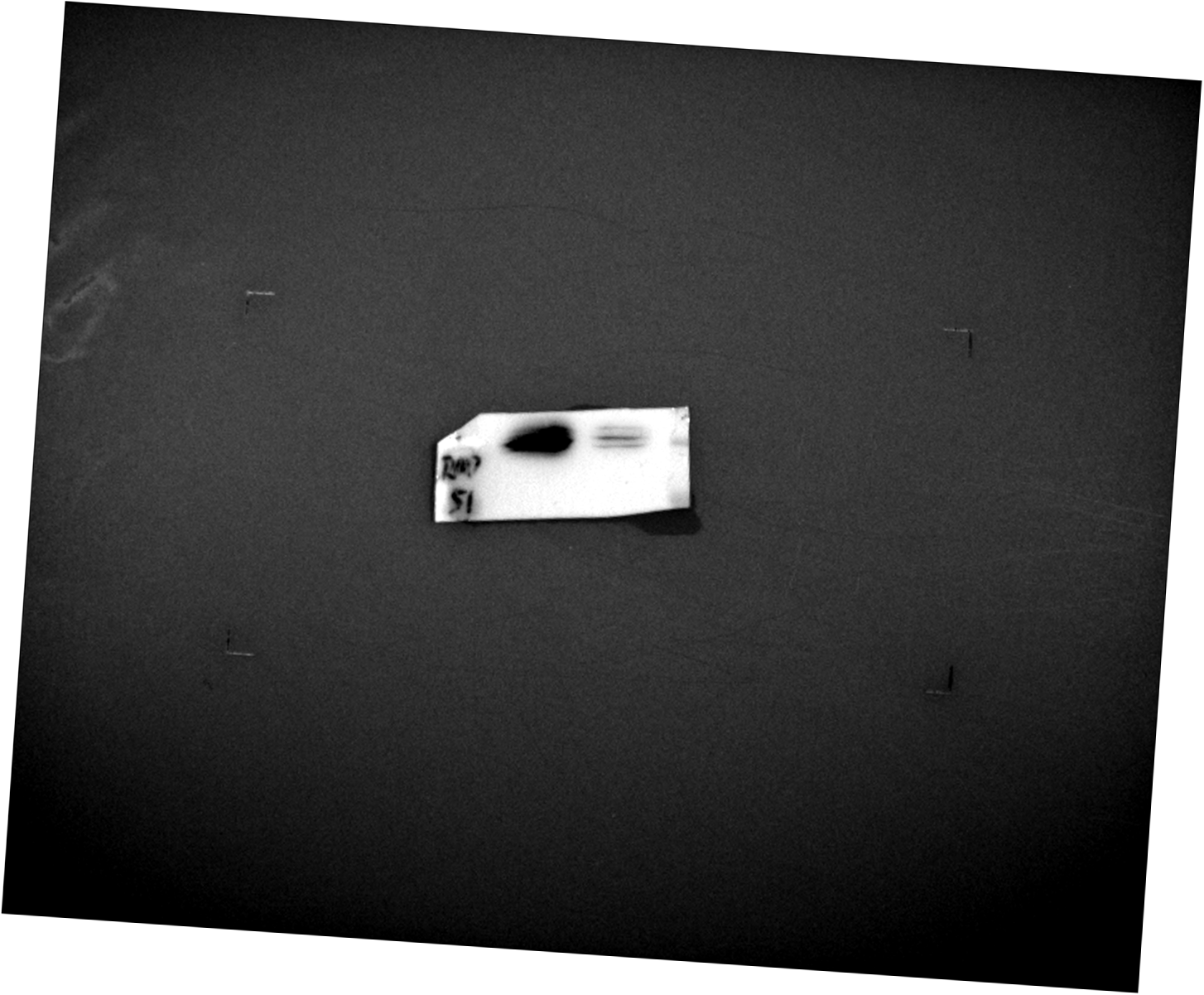

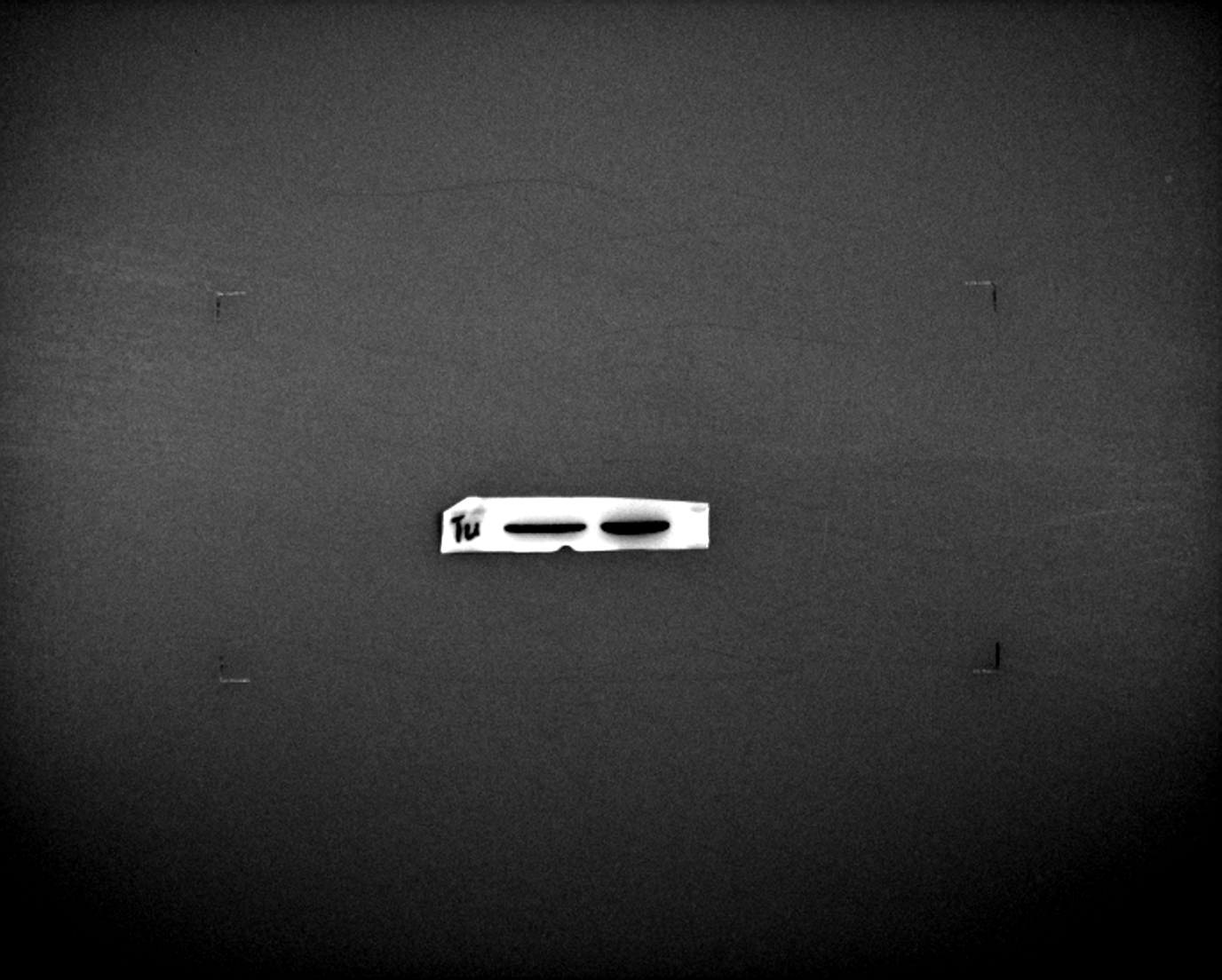

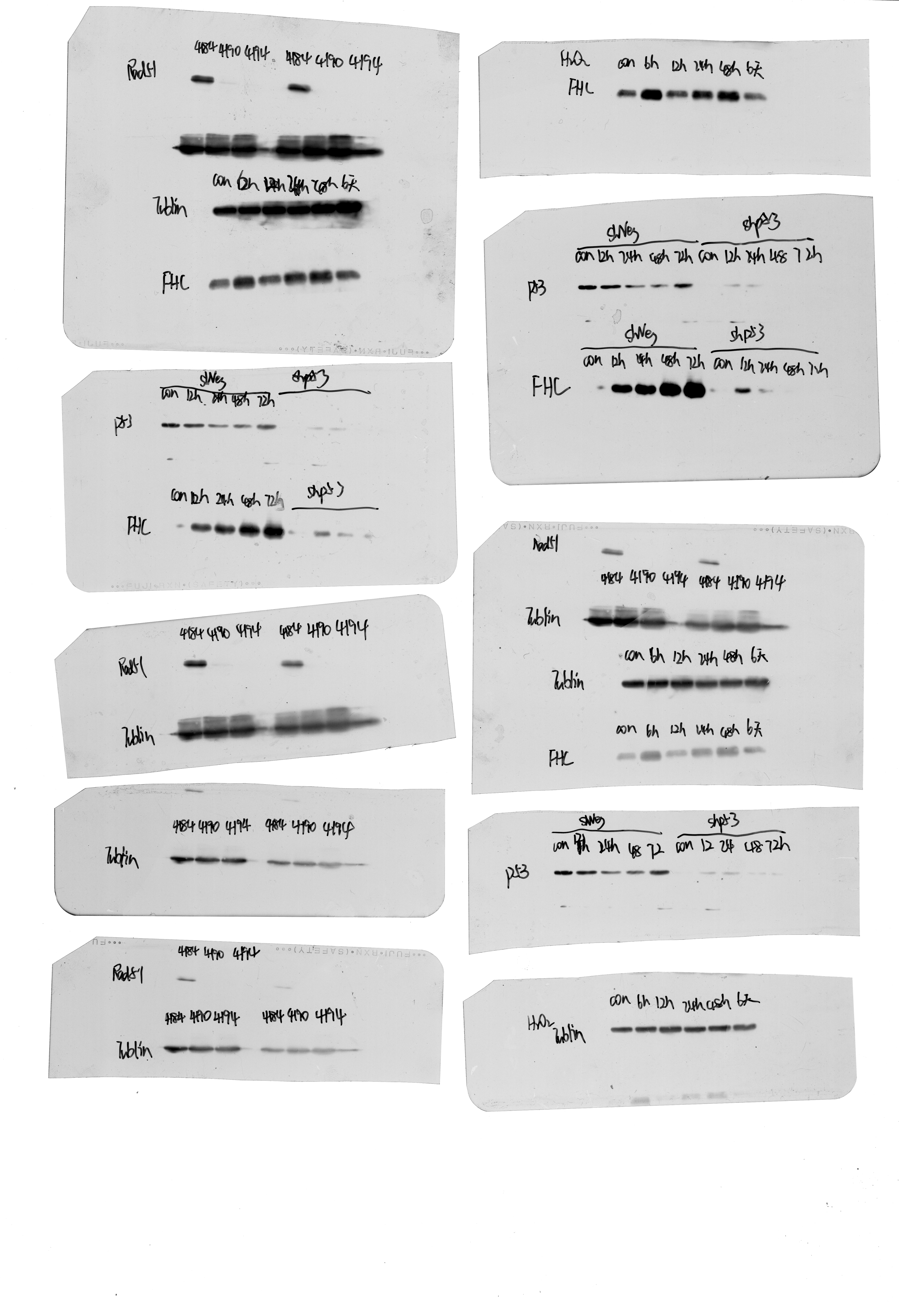

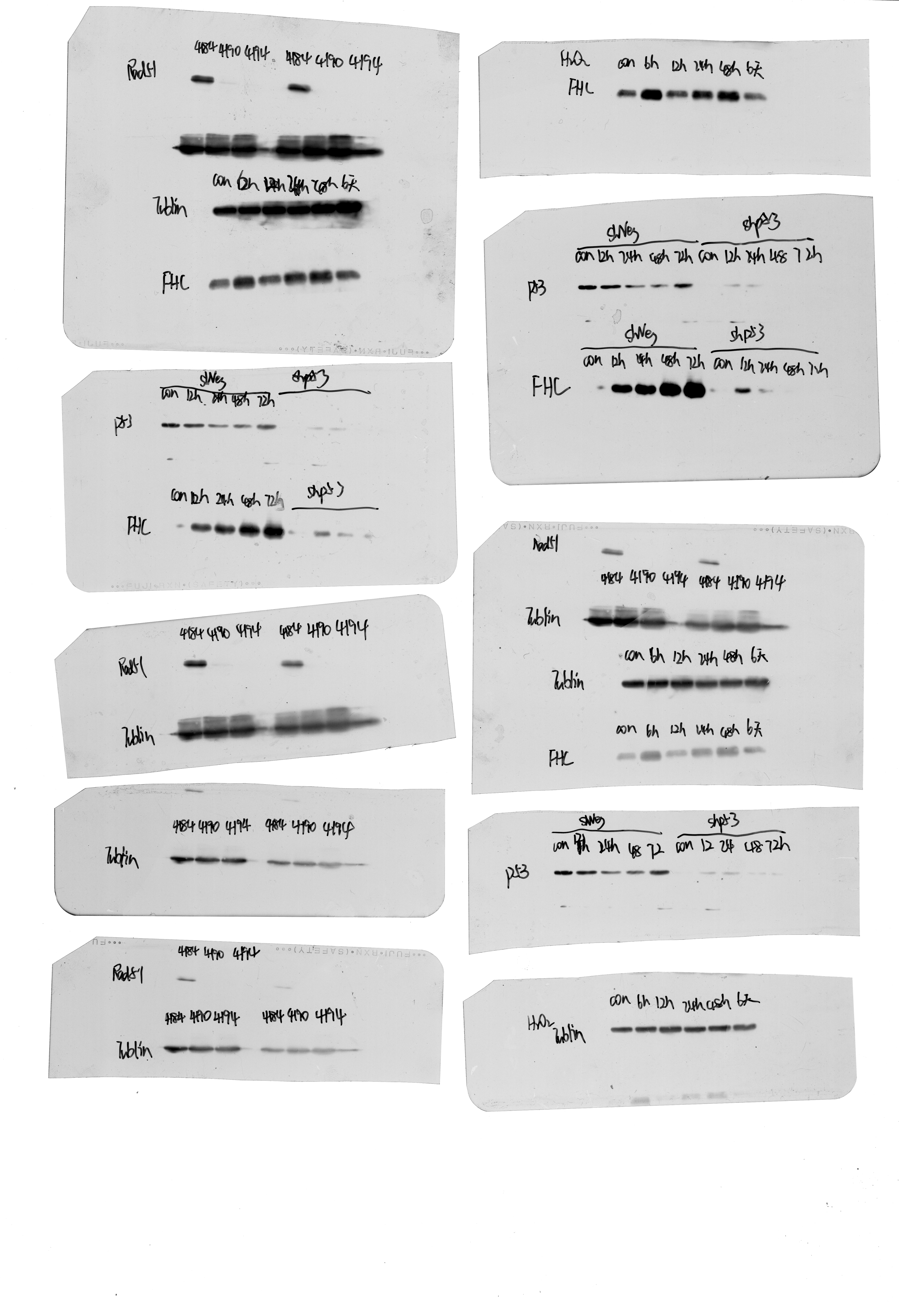

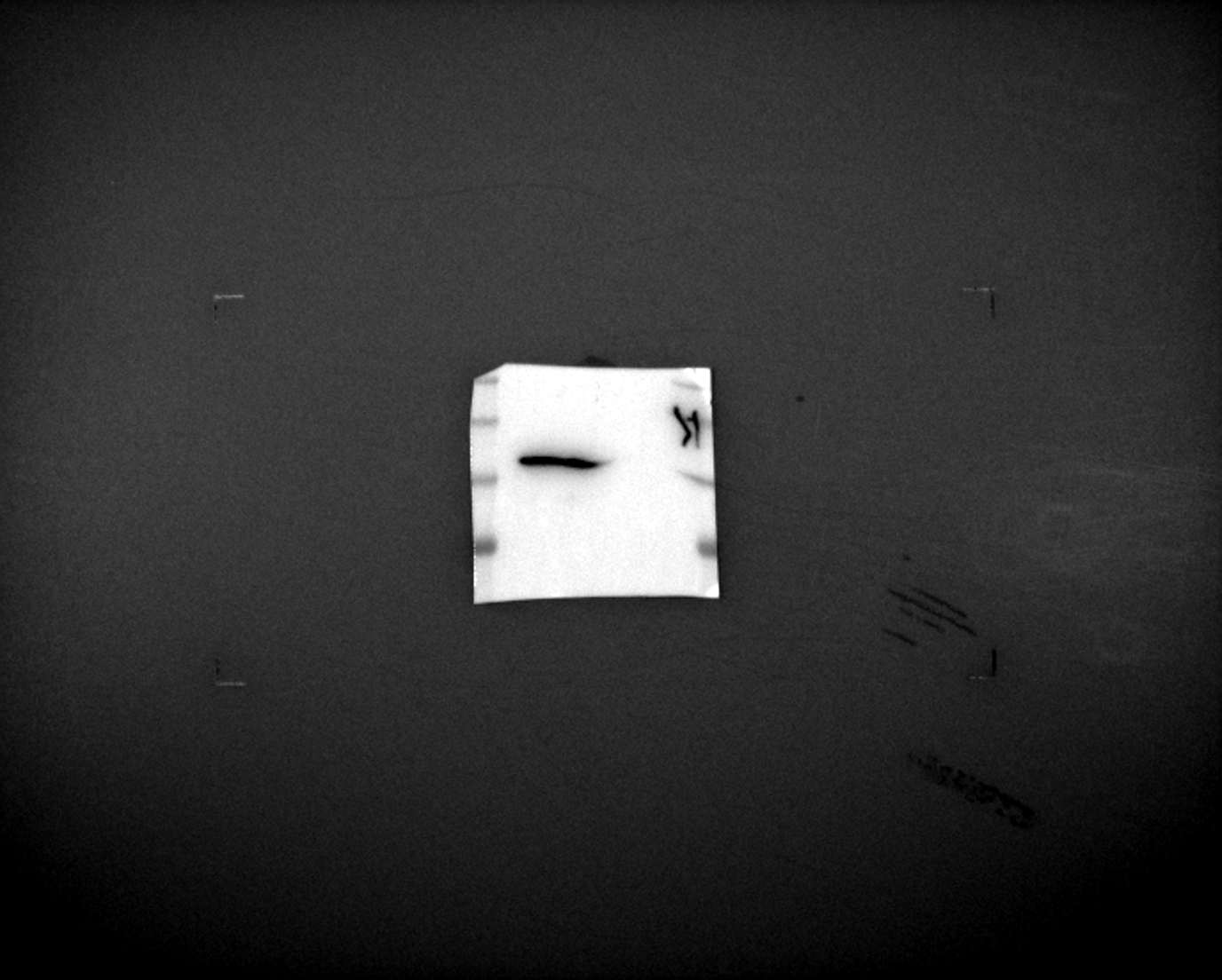

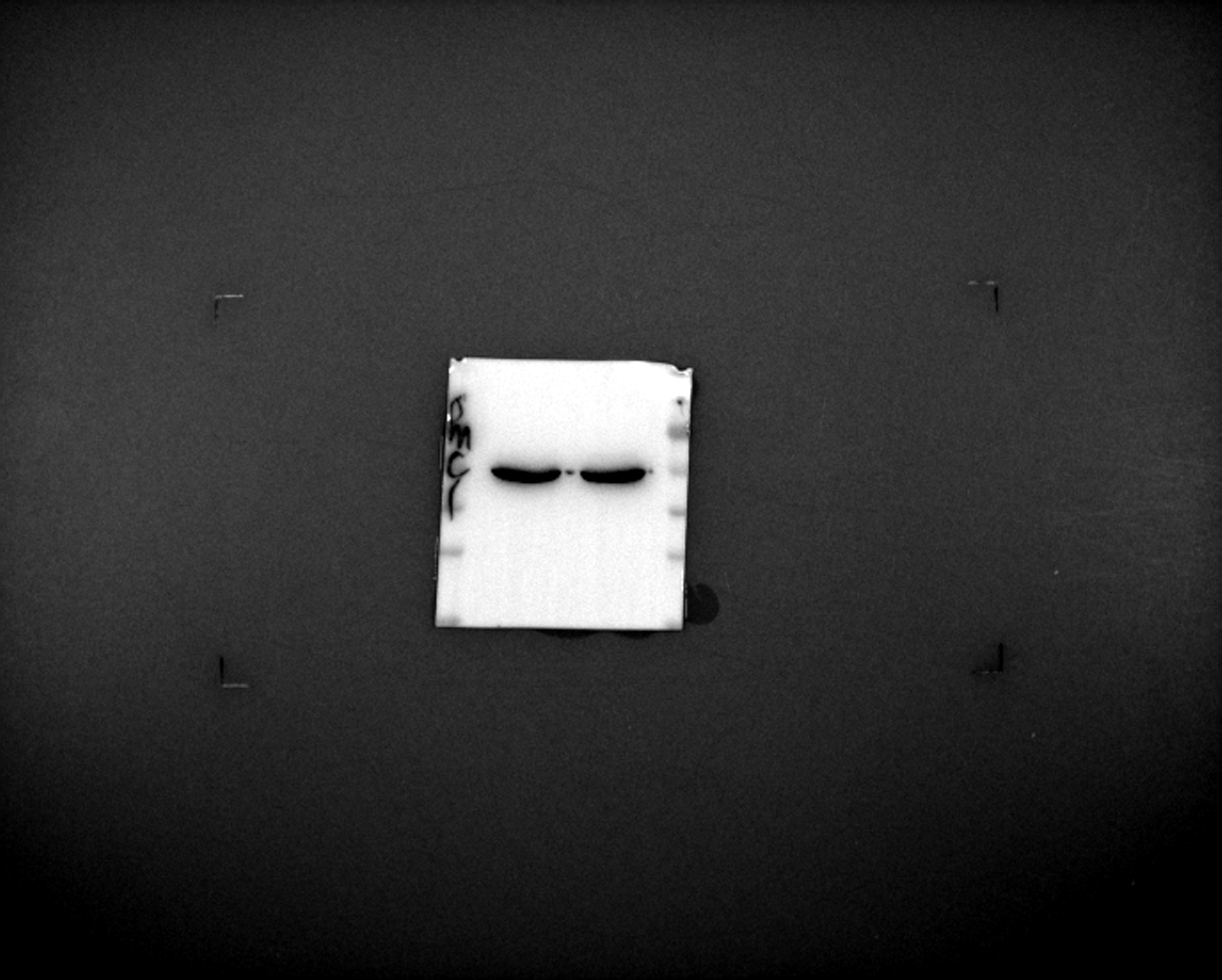


35kDa

25kDa

45kDa

35kDa

25kDa

45kDa

GAPDH

RAD51

GAPDH

35kDa

45kDa

35kDa

25kDa

45kDa

RAD51

Figure 1

Liver

Spleen

Lung

Kidney

Ovary

Testis

Brain

PD6

PD8

PD10

PD12

PD14

PD20

PD25

Adult

1A

1B

135kDa

100kDa

180kDa

75kDa

MVH

FSPCs

FSCs

25kDa

35kDa

45kDa

RAD51

25kDa

35kDa

45kDa

DAZL

45kDa

65kDa

Tubulin

1D

Figure 2

2C

RAD51

Tubulin

35kDa

45kDa

65kDa

55kDa

Control

*VKO*

*VKO*

Figure 4

4D

25kDa

35kDa

45kDa

55kDa

25kDa

35kDa

45kDa

55kDa

70kDa

100kDa

RAD51

Tubulin
